# A benchmark of individual auto-regressive models in a massive fMRI dataset

**DOI:** 10.1162/imag_a_00228

**Published:** 2024-07-15

**Authors:** François Paugam, Basile Pinsard, Guillaume Lajoie, Pierre Bellec

**Affiliations:** University of Montréal, Montréal, Canada; Centre de Recherche de l’Institut Universitaire de Gériatrie de Montréal, Montréal, Canada; Mila - Institut Québécois d’Intelligence Artificielle, Montréal, Canada

**Keywords:** fMRI, auto-regression, graph convolution, dense sampling, deep learning, individual brain models

## Abstract

Dense functional magnetic resonance imaging datasets open new avenues to create auto-regressive models of brain activity. Individual idiosyncrasies are obscured by group models, but can be captured by purely individual models given sufficient amounts of training data. In this study, we compared several deep and shallow individual models on the temporal auto-regression of BOLD time-series recorded during a natural video-watching task. The best performing models were then analyzed in terms of their data requirements and scaling, subject specificity, and the space-time structure of their predicted dynamics. We found the Chebnets, a type of graph convolutional neural network, to be best suited for temporal BOLD auto-regression, closely followed by linear models. Chebnets demonstrated an increase in performance with increasing amounts of data, with no complete saturation at 9 h of training data. Good generalization to other kinds of video stimuli and to resting-state data marked the Chebnets’ ability to capture intrinsic brain dynamics rather than only stimulus-specific autocorrelation patterns. Significant subject specificity was found at short prediction time lags. The Chebnets were found to capture lower frequencies at longer prediction time lags, and the spatial correlations in predicted dynamics were found to match traditional functional connectivity networks. Overall, these results demonstrate that large individual functional magnetic resonance imaging (fMRI) datasets can be used to efficiently train purely individual auto-regressive models of brain activity, and that massive amounts of individual data are required to do so. The excellent performance of the Chebnets likely reflects their ability to combine spatial and temporal interactions on large time scales at a low complexity cost. The non-linearities of the models did not appear as a key advantage. In fact, surprisingly, linear versions of the Chebnets appeared to outperform the original non-linear ones. Individual temporal auto-regressive models have the potential to improve the predictability of the BOLD signal. This study is based on a massive, publicly-available dataset, which can serve for future benchmarks of individual auto-regressive modeling.

## 1 Introduction

Auto-regressive models serve as fundamental tools to understand the intricate dynamics of brain activity in functional magnetic resonance imaging (fMRI). Traditionally, fMRI auto-regressive models have focused on linear space-time interactions at the group level, often constrained by limited recording time per subject (typically less than 30 min). The emergence of dense functional neuroimaging datasets, providing extensive recordings for each individual, has opened up new possibilities. By harnessing individual auto-regressive models with dense samples, it becomes feasible to unveil potential non-linear interactions that might be obscured by group-level averaging. In this study, we capitalize on an ultra-dense individual fMRI dataset to compare the accuracy of various types of temporal fMRI auto-regressive (AR) models. These models encompass both linear and non-linear architectures, including a variety of deep neural networks.

AR models of fMRI data are used to capture temporal auto-correlations in brain dynamics: past values of the fMRI time-series are used to predict the future values. This type of modeling can be used to infer the functional interactions between brain regions, notably using Granger Causality (Seth et al., 2015). AR models can also be used to whiten the residuals in general linear models of brain activity (Bullmore et al., 1996). They have also been proposed as possible foundation models trained on a very large collection of fMRI samples, in order to train effective brain decoding models as a downstream task using limited amounts of data (Thomas et al., 2023). Although AR models based on fMRI data only indirectly probe neural processes through their vascular correlates, they have been proposed as a reasonable proxy to study functional brain dynamics (Lurie et al., 2020;Matsui et al., 2019;Rogers et al., 2010). Because of these many applications, and the pivotal role of general linear models in particular, AR models are ubiquitous in the fMRI literature. For this reason, a number of studies have focused on auto-regression itself and examined the performance of various AR techniques in terms of its core objective: the accuracy of future time point prediction (e.g.,Garg et al., 2011;Wein et al., 2021,^2022^). The classical approach to temporal auto-regression is a vector auto-regressive (VAR) model, where a linear multivariate regression is applied at the group level, typically looking at a single time point in the past to predict the next time point, using identical model parameters for all subjects (Seth et al., 2015). Similarly, the standard methods for functional connectivity rely on the linear correlations between brain regions (Hlinka et al., 2011). Thus, the methodology for studying the functional organization of fMRI brain data is dominated by linear assumptions. Even though these linear assumptions have been shown to be suitable in group studies with a limited amount of data per subject (Hlinka et al., 2011;Rogers et al., 2010), recent work using graph convolution networks has shown better modeling performance of non-linear models with a massive group sample collected by the Human Connectome Project (Wein et al., 2021). Non-linear models appear especially interesting as their higher expressivity enables them to capture complex state-transition dynamics. The potential benefits of complex non-linear models are, however, critically dependent on the type and the size of datasets, and may only become apparent with very large datasets. For example,Schulz et al. (2020)showed that non-linear deep neural networks scale in performance with increasing dataset sizes on a number of different machine-learning tasks in the UK biobank sample. Non-linear models also clearly outperformed linear models in brain decoding of the human connectome project tasks (Zhang et al., 2022), yet the same human connectome task benchmark led to different optimal architectures when applied on deep individual fMRI data (Rastegarnia et al., 2022).

In this study, we propose to study the behavior of auto-regressive models in the context of the ultra-dense dataset collected by our team as part of the Courtois project on neuronal modeling (Courtois NeuroMod) (Boyle et al., 2020). We trained the models on the so-called Friends dataset, where subjects watched the friends TV show for two seasons (roughly 20 h of data per subject). We selected a naturalistic stimulus because it enables collecting a massive amount of fMRI data while keeping the subject engaged, which has shown to lead to improved test-retest reliability of fMRI connectivity (Wang et al., 2017). Both intrinsic and extrinsic fluctuations are present in any experimental condition, whether task-oriented or rest, so we expect auto-regressive models to be able to capture fluctuations evoked by the movie and shared across subjects, along with idiosyncratic or spontaneous brain activity. To further test how this type of model would generalize to different types of extrinsic and intrinsic activity, we also examined how the auto-regressive models generalized both to resting-state (30 min per subject) and other types of naturalistic stimuli (four different full movies, about 10 h of fMRI per subject). Taken together, the scale and diversity of the stimuli used in this individual benchmark is unprecedented, leveraging several large datasets collected by Courtois NeuroMod on the same individuals.

Our first aim for this study was to compare standard multivariate and univariate linear models to several deep neural networks of various architectures on a temporal AR task; seeTable 1. Each model is subject-specific, and evaluated at six different time lags. The auto-regression task performed by the models consists in predicting the activity vector at timet+lfrom the activity vectors from timet−ktot. The main characteristics of the 11 different model architectures used in this benchmark are presented inTable 1. The best performing architecture, a graph neural network, was subjected to a sensitivity analysis to identify the importance of its design features, such as the choice of the graph and the presence of non-linearities. Our second aim was to quantify the data scalability of the best model architecture identified in aim 1, as well as the scalability of classical, linear models. Our third aim was to characterize the extent to which individual models of the selected architecture were indeed subject-specific. This was achieved by comparing the performance scores of individual AR models on data coming from different subjects to the individual used for model training, as well as a group model trained on multiple subjects at the same time. Our fourth aim was to study the spatiotemporal structure of the signals captured by the best performing auto-regressive model (from aim 1), and to assess if this model recovered known physiological characteristics previously reported with classical functional connectivity analyses. Our fifth and final aim was to assess whether the best performing models generalized to different types of extrinsic and intrinsic activity. We first applied the models to a series of new datasets (resting-state and different movies). We then tested the auto-regressive models on movie watching fMRI data with the shared response (between subjects) regressed out.

**Table 1.** Main characteristics of the models used in the benchmark.

Model	Chebnet	LR uni	MLP uni	LR multi	MLP multi	RNN	NBEATS	GRU	LSTM	TFT	Transformer
linear		X		X							
univariate		X	X								
multivariate				X	X	X	X	X	X	X	X
graph convolution	X										
recurrent						X		X	X	X	
attention										X	X
input length	192	256	128	3	3	64	2	64	32	32	1
number of parameters	8e3	5e4	2e5	1e5	6e5	2e4	5e5	1.5e4	2e5	7e5	3e5
training time (min)	13	3	4	1	1	6	3	2	2	73	4
size of gridsearch	630	196	784	42	168	288	576	288	288	12	96

The input length, number of parameters, and training times correspond to the models with the best hyper-parameters identified through the grid searches. The training times reported were obtained using a Quadro P5000 GPU, and correspond to the training for one subject.

Taken together, the aims of this work should clarify under which conditions individual auto-regressive models can be effectively trained in the context of a massive dense fMRI dataset, in terms of model architecture, dataset size, and type of experimental conditions.

## 2 Methods

### 2.1 Datasets

The datasets used in this study come from the Courtois NeuroMod databank (Boyle et al., 2020). The datasets were acquired on 6 subjects (3 women, 3 men), all right handed and from 31 to 49 years of age at acquisition. Three participants reported being native francophones, one reported being a native Anglophone, and two reported being bilinguals. All participants have a solid comprehension of the English language and reported watching English TV. All stimuli were presented in English. All subjects provided informed consent to participate in this study, which was approved the ethics review board of the “CIUSS du centre-sud-de-l’île-de-Montréal” (under number CER VN 18-19-22).

fMRI data were collected using a 3T Siemens Prisma Fit scanner, and an accelerated simultaneous multi-slice imaging sequence (Setsompop et al., 2012). The spatial resolution is 2 mm isotropic, and the TR is 1.49 s. Data were preprocessed using the fMRIprep pipeline (Esteban et al., 2019), using the “long term support” version 20.2.3. The BOLD time-series registered into the MNI space (MNI152NLin2009cAsym) were projected on the MIST atlas (Urchs et al., 2019) at the scale of 197 parcels. The estimated motion parameters and the global signal were regressed out and a high-pass filter at 0.01 Hz was applied, using a discrete cosines basis. The time-series of each parcel were normalized per run to a zero mean and unit variance.

*friends dataset*(about 9 h 50 min). Subjects watched the seasons 1 (approximately 9 h 30 min of data) and 2 (approximately 9 h 20 min of data) of the television series friends while being scanned. The two seasons total about 19 h of fMRI data.

The season 1 and 2 of the*friends*dataset were used for the training, validation, and testing of all models. Season 1 was used as the training set (about 9 h), and season 2 was divided into the validation and test sets by separating even and odd episodes respectively (about 4 h 40 min each). For sub-02 to sub-06, this resulted in 22,267 volumes for training, 11,224 for validation, and 11,358 for test. For sub-01, the data of several episodes were not available, which resulted in 21,313 volumes for training, 8,500 for validation, and 8,508 for test.

The*hcp-trt*and*movie10*dataset, both described below, were used to evaluate the generalisability of the models to data acquired while subjects were performing other tasks.

*hcp-trt dataset*(utilized 75 min). Subjects did 15 repetitions of various short (under 5 min) cognitive tasks from the Human Connectome Project (Van Essen et al., 2012). Additionally 5 repetitions of the resting-state task (15 min x 5 runs = 75 min) were acquired on each subject. Given that the models evaluated for task generalization require long input sequences (∼5 min), only the resting-state tasks were used.

*movie10 dataset*(∼10 h). The movie10 dataset corresponds to scans during which the subjects watched movies (*The Bourne Supremacy, Life, Hidden Figures*and*The Wolf of Wall Street*) split in∼10 min long segments while being scanned. Participants watched*Hidden Figures*and*Life*twice.

In addition to the functional data, the preprocessing pipeline (sMRIPrep) used T1- and T2-weighted anatomical scans for segmentation, surface reconstruction, and registration to standard templates. For more information on the anatomical data, sequence parameters, scanner setup, and each of the datasets, please consult the Courtois Neuromod’s Project documentation page: (www.docs.cneuromod.ca/en).

### 2.2 Models

The auto-regressive task that each model is performing can be formalized as solving the following equation:

$$X_{t+l}=f\left(\left\{X_{i}|t-k<i\leq t\right\}\right)+{\#}_{t+l}$$ (1)

whereXis the BOLD time-series in the parcel space (i.e., the signal in a parcel is the average signal of the voxels in the parcel),tthe time index,lthe lag between the predicted time point and the last time point in the input,fthe model,kthe number of past time points used as input, and#the error term to minimize. In other words, the model must predict the values of the signal at time pointt+l, given the values at time pointst−ktot. In this study, except during the hyper-parameter search, we focused on models only trained for prediction at lagl=1. Using these, we evaluated performance at lagsl=1to 6 TRs (i.e., 1.49 s to 8.94 s), where predictions at lags greater thanl=1consist in reiterating the prediction process with the previous predictions used as inputs. For example, for prediction at lagl=3, the model will use the true values at time pointst−k+2totand the predicted value fort+1andt+2.

The different types of models used in this study are the following:

- linear multivariate and univariate models, with the multivariate linear models corresponding to the classical VAR models. The predicted value for a parcel is a linear combination of the past values of only this parcel (univariate) or of all the parcels (multivariate). The multivariate models can model spatial autocorrelation, whereas the univariate cannot;
- multivariate and univariate multi-layer perceptrons (MLPs) which correspond to stacked linear models with non-linear activation functions (here the rectified linear unit);
- the Chebnet (Defferrard et al., 2017), a type of graph convolutional neural network (Z. Wu et al., 2021). Graph convolutions are an extension of standard convolutions to non-grid-like graphs. Local interactions between nodes are modeled as convolution filters applied to each node’s neighborhood. The graphs used for the graph convolutions have all the parcels as nodes, and their binary connections are the 10% of the pairs of parcels with the highest functional connectivity. The functional connectivity is computed for each subject as the Pearson correlation between the parcels on the training data. The functional connectomes provided to these models constitute strong priors on the spatial autocorrelation of the time-series. For a graph sensitivity analysis, we also trained models with binary random and spatial connectomes with the same graph density of 10%. The spatial connectome was generated from the minimal euclidean distance of voxels from pairs of parcels;
- simple recurrent neural networks (RNN), which keep a representation of the current state of the time-series as an internal state vector;
- the long short term memory network (LSTM) (Hochreiter & Schmidhuber, 1997), a more elaborate kind of RNN which can better control what information to retain in the state representation through the use of gates;
- the gated recurrent unit network (GRU) (Chung et al., 2014), another kind of RNN using gates, but simpler gates than the LSTM;
- the neural basis analysis for the time-series model (N-BEATS) (Oreshkin et al., 2020), a stack of fully connected neural network blocks, each modeling the residuals of the other blocks;
- the Transformer (Vaswani et al., 2017) which uses attention heads to focus on the relevant interactions in the input space;
- the temporal fusion transformer (TFT) (Lim et al., 2020) which is a variation on the transformer, with the addition of recurrent units.

For the RNNs, GRUs, LSTMs, N-BEATS, Transformer, and TFT, the implementations from the python library Darts (Herzen et al., 2022) were used. The other models were implemented by us using the pytorch library (Paszke et al., 2019). In particular, our implementation of the Chebnet uses torch_geomtric’s chebyshev spectral graph convolution (ChebConv) module.

### 2.3 Hyper-parameter search

For each type of model, a grid search was performed to identify the best hyper-parameters. The models were trained independently for each subject, and the hyper-parameters were optimized for the mean score over subjects. The best hyper-parameters were not identified in a subject-specific manner to have more consistent comparisons across subjects, and to identify the overall optimal architecture for the auto-regresssive task, which then can be potentially applied to other subjects.

For all models, the Adam optimizer (Kingma & Ba, 2017) was used, without L2 regularization. For the gridsearch, a single training / validation split was used for each subject: the models were trained on the training set (*friends*season 1,∼9 h) and evaluated on the validation set (half of season 2,∼4 h40). For all models from the darts library, the grid search was centered around the default values provided by the library. The scope of all grid searches is provided in theSupplementary Material, section V.

For the Chebnets, the univariate and multivariate linear models and MLPs, during the grid search the models were trained independently on tasks of lagl=1tol=6, meaning that for each lag and subject, a model was trained. The selection of the best hyper-parameters was based on the average score, across subjects, parcels, and lags. For the recurrent models (RNNs, LSTMs, and GRUs), due to their implementation, the prediction at a lagl>1is done iteratively with the intermediate predictions fed back to the models as new inputs. Thus for these models, during the hyper-parameter search, the training task was always the prediction at lagl=1, but the evaluation criteria were the average prediction score at lagsl=1tol=6. For the N-BEATS, Transformer, and TFT models, during the hyper-parameter search, the models were trained to predict lags ranging froml=1tol=6. To have a fair comparison of the models performances, all the scores presented inFigure 1correspond to models trained on the task with only lagl=1(using the best hyper-parameters found in the grid searches), with the predictions at lagsl>1obtained recursively. This way the prediction method, although slightly different in the hyper-parameter search, is consistent for the model comparison. Moreover, the subsequent analysis of the best performing models is also only pertaining to the models trained for prediction at lagl=1.

**Fig. 1.**
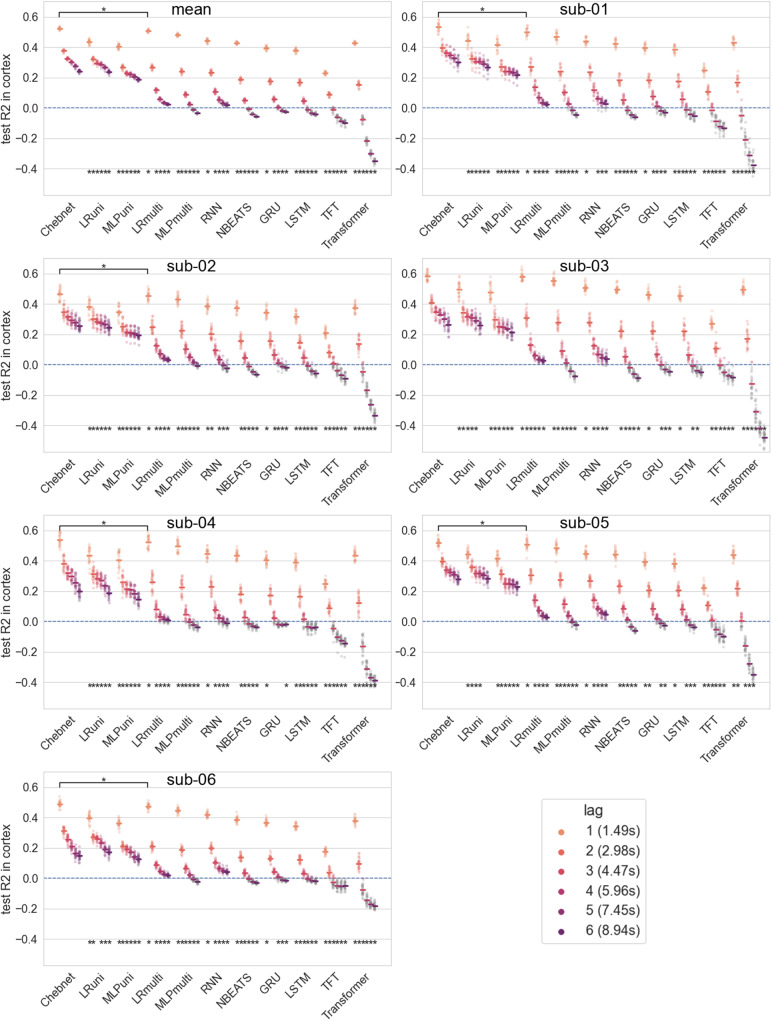
Accuracy ($R^{2}$) score of auto-regression prediction models at different time lags averaged over all cortical brain parcels. Top left is the mean over all subjects, remaining panels are individual subjects. The Chebnet is overall the model architecture that explains the most amount of variance, with the linear multivariate coming close second on lagl=1, and the linear univariate on lagl=5andl=6. Dots are the scores per run, rectangles are the averages on runs per model and lag. Negative$R^{2}$scores of runs are grayed out, as a negative$R^{2}$signifies that the model’s prediction is worse than a naive model that would always predict the mean of the signal. The bottom asterisks denote a significant difference between the$R^{2}$scores of the model and lag and the scores of the previous model (next one to the left) for the same lag. The asterisks at the top correspond to the same test for the difference between the scores of the Chebnet and the linear multivariate for lagl=1. The significance corresponds to a two-sided Wilcoxon test (p<0.05).

The performance metric (loss function) used to train the models is the mean squared error between the predicted and true signal. The best hyper-parameters were selected based on the$R^{2}$score between the true signal and the predicted one, which is the metric reported in the results. The$R^{2}$score corresponds to the proportion of variance of the true signal explained by the predicted signal. For the hyper-parameter search, the$R^{2}$was averaged across all parcels, but in most of the results presented here, the$R^{2}$is averaged across the parcels that are in the cortex.

### 2.4 Shared response

A common method to estimate the evoked response consists in computing the shared response by averaging the activity of multiple subjects exposed to the same stimulus (Nastase et al., 2019). We estimated the intrinsic activity in a leave-one-subject-out manner. For each subject, we computed the shared response on the other subjects and regressed out this shared response from the subject’s BOLD signal. Therefore, the intrinsic activity is modeled as the residuals of the shared response. For the model architecture yielding the highest accuracy scores on the whole BOLD signal, we trained similar models on the intrinsic activity of each subject. We then compared the performances of the models trained and evaluated on the whole BOLD signal to the performances of the models trained and evaluated on the intrinsic activity.

### 2.5 Seed-based connectivity maps

A seed-based connectivity map represents the functional connectivity between one seed region and all of the voxels recorded in the brain. Here, we measure the functional connectivity as the Pearson correlation, thus our seed-based connectivity maps show which voxels of the brain correlate with the signal in the seed parcel. The maps presented in the results are using seeds in 6 of the 7 Yeo networks (Thomas Yeo et al., 2011): the default-mode network with a seed in the cingulate gyrus (-7, -52, 26), the visual network with a seed in the superior lingual gyrus (-16, -74, 7), the sensorimotor network with a seed in the postcentral gyrus (-41, -20, 62), the dorsal attentional network with another seed in the postcentral gyrus (-34, -38, 44), the ventral attentional network with a seed in the cingulate gyrus (-5, 15, 32), and the fronts-parietal network with a seed in the middle frontal gyrus (-40, 50, 7). The seventh limbic network was excluded because it is composed of regions with high signal loss and distortions due to field inhomogeneity. For each seed, the signal in the corresponding parcel (original or predicted) was used to compute its correlation to the original signal of all the voxels.

### 2.6 Statistical significance

Every statistical significance reported corresponds to a p-value inferior to 0.05, with a False Discovery Rate correction applied for multiple comparison, following the Benjamini-Hochberg procedure.

## 3 Results

### 3.1 The Chebnets are the best performing models, with linear models as close seconds

We first compared different auto-regressive models to identify which performed best on parcel-wise averaged BOLD time-series auto-regression in the friends dataset. We also assessed auto-regressive tasks for varying time horizons (lags), that is, predicting brain activity more or less distant in the future. We compared (seeFig. 1) the mean cross-validated accuracy score of each type of model at different prediction lags, ranging from 1 to 6 TRs (1.49 s to 8.94 s). The Chebnets have the best overall performance for every prediction lag. The linear multivariate model came as a close second on lagl=1, and the linear univariate came second for lagsl=2to 6, with close scores for lagsl=5andl=6. All other models (except the TFT) had similar performance for lagsl=1andl=2. This behavior was consistently observed on all six participants. We conclude that, at least with this amount of 9 h of training data, the only architecture that presents an advantage over linear models is the graph convolution Chebnet, which is also the only model to perform well at both short and long lags.

### 3.2 Graph sensitivity: using the functional connectome for graph convolutions improves the accuracy at shortest lag, compared to spatial or random connectomes

We conducted a sensitivity analysis on the graphs provided to the Chebnet models regarding the type of graph prior used in the model. The graphs used in the comparison to other model architectures are binarized functional connectomes, which are expected to provide a good prior for modeling spatial autocorrelation of the brain dynamics. We compared the accuracy of models trained using the binarized functional connectome, to models using random binary graphs and binarized spatial connectomes with the same density of edges. This comparison is displayed onFigure 2. The accuracy of the models with the functional connectomes appeared significantly superior to the two other kinds of graphs for all subjects for the lagl=1. For longer lags, the difference in accuracy decreases with increasing lag, with the spatial and random graphs occasionally significantly outperforming the functional one for some subjects, while the spatial graph generally outperformed the random graphs.

**Fig. 2.**
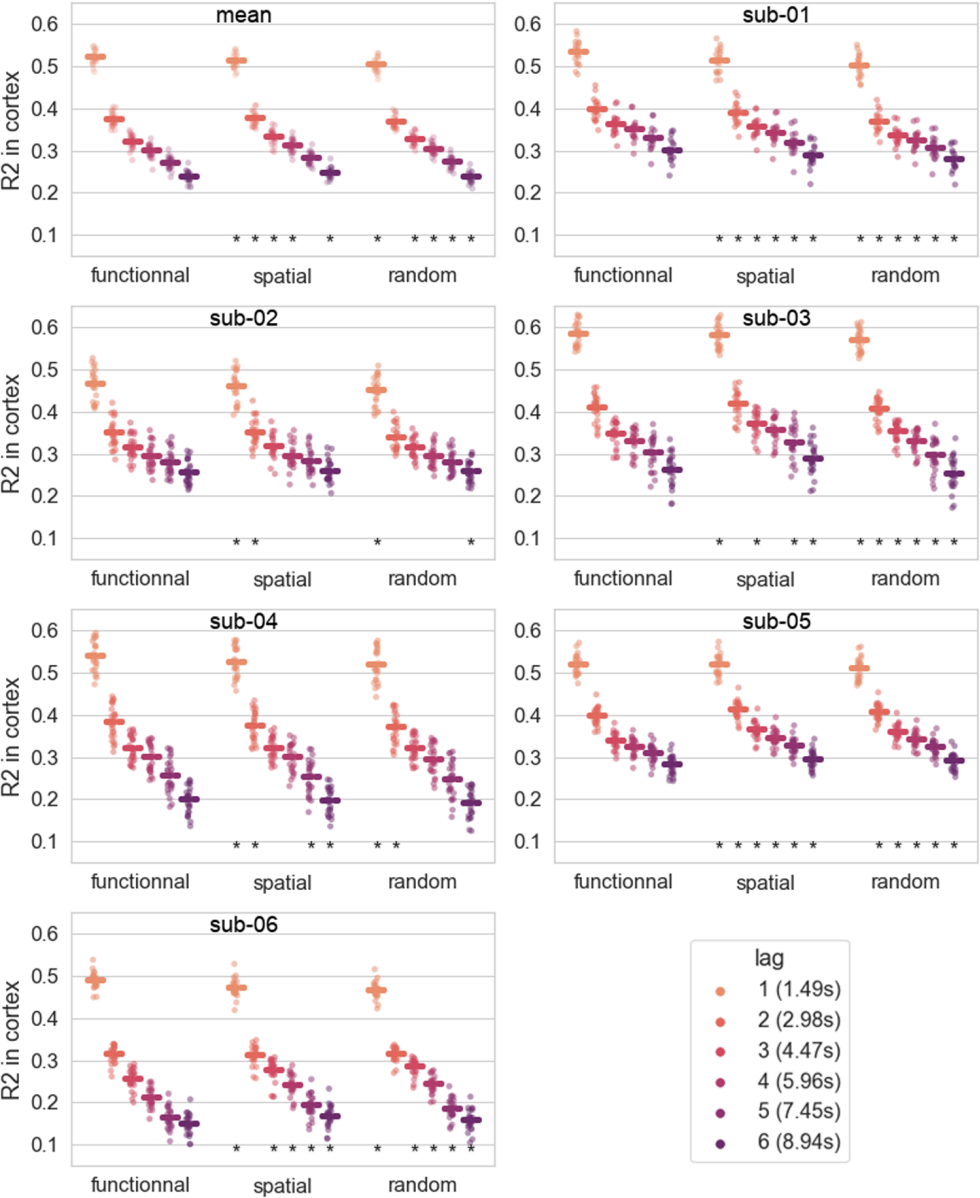
Graph sensitivity. Accuracy ($R^{2}$) score of Chebnet models trained with either functional, spatial, or random graphs; all with the same graph density (10%). The scores for the random graphs correspond to the average score over 10 trials with different random binary graphs. The asterisks denote a significant difference with the corresponding scores of the functional graph’s model, from a two-sided Wilcoxon test (p<0.05). The functional graphs give significantly better accuracy for all subjects at lagl=1. This advantage diminishes and disappears with increasing lags, with spatial or random graphs occasionally outperforming the functional one depending on the subject.

### 3.3 Non-linearity sensitivity: removing the non-linearities of the Chebnets improves their accuracies

We conducted a non-linearity sensitivity analysis by comparing the Chebnets to linear versions of the Chebnets. The linear Chebnets have identical architectures, except for the non-linear Rectified Linear Unit activations functions which were removed in the linear version of the Chebnets. Surprisingly, the accuracy of the linear Chebnets was significantly higher than the accuracy of the non-linear ones for almost all subjects and lags (seeFig. 3).

**Fig. 3.**
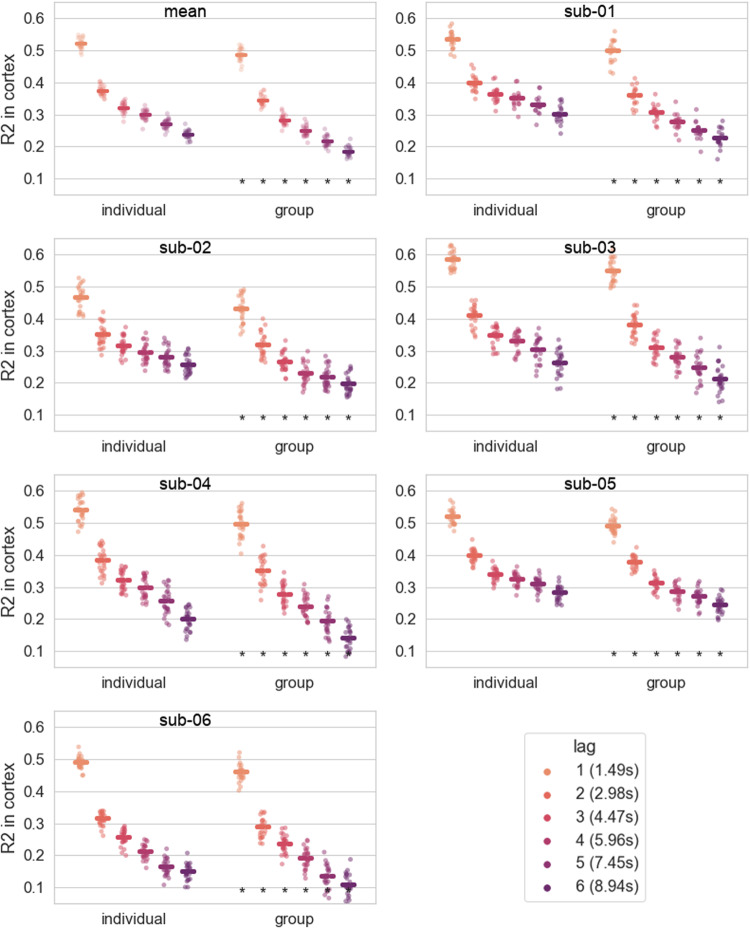
Non-linearity sensitivity analysis. Comparison of the prediction accuracy scores ($R^{2}$) between non-linear Chebnets and linear Chebnets. Asterisks denote the significance of a two-sided Wilcoxon test between the$R^{2}$scores of linear and non-linear models for a specific lag. Linear Chebnets have significantly better prediction accuracy for all lags and subjects, except sub-01 lagl=1.

### 3.4 The Chebnets accuracy scales with the amount of training data, and does not reach saturation even with 9 h of training data

We wanted to clarify whether a massive amount of fMRI data is required in order to train auto-regressive models using Chebnets. We trained Chebnets using the same hyper-parameters as those previously identified with the grid search, on varying training sets of increasing sizes. For each subject, the Chebnet was trained from scratch with an increasing number of acquisition runs, by increments of 4 runs from 4 (∼45 min) to 44 (∼9 h). The training was repeated five times for each amount of runs. Each model is evaluated on the same validation dataset as the one from the hyper-parameter search. The mean evaluation scores are reported onFigure 4and show that the performance keeps increasing with the amount of data, with no clear plateau at the maximum value of data (9 h). This observation applied to most time lags in all subjects, and was particularly clear for the shortest time lag, with the longest time lags showing less pronounced slopes. These results demonstrate that large amounts of data are required to train individual auto-regressive Chebnet models with high accuracy, and that the dataset we used (friends season 1, 9 h of fMRI data per subject) was not sufficient to saturate the performance of the model. Similar trends were found for the linear multivariate and univariate models; the corresponding figures are presented in theSupplementary Material, section III.

**Fig. 4.**
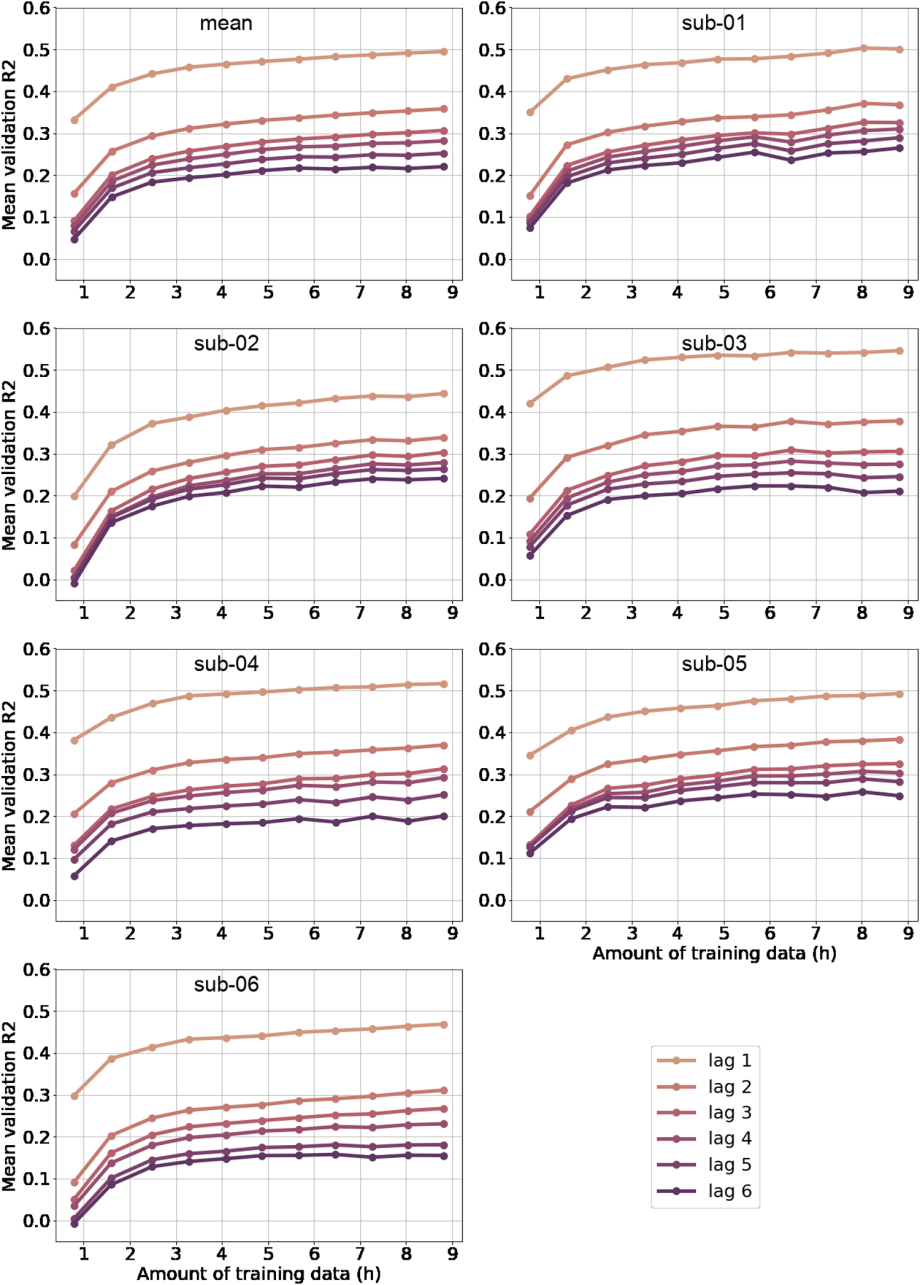
Effect of training data amount on validation$R^{2}$of the Chebnets. The performance of the model increases with more training data, with no clear plateau.

### 3.5 Subject specificity decreases with increasing lags

All the models for this study being individual (meaning each model is independently trained and evaluated on the data from one subject), we looked at the subject specificity of the Chebnet models. In order to do that, we compared the intra-subject and inter-subjects$R^{2}$, averaged across parcels. The intra-subject$R^{2}$scores are computed by evaluating the model on the test data of the subject it was trained on (seeFig. 5a). The inter-subject$R^{2}$scores are computed by evaluating the model on the test data of other subjects than the one it was trained on. A model is considered subject-specific if its intra-subject scores are significantly higher than its inter-subject scores, which means that it is better at modeling the data of the subject it was trained on than other subjects’ data. We thus looked at the difference between intra- and inter-subject$R^{2}$for each pair of subjects, at different lags (seeFig. 5b). At lagl=1all the models are significantly subject-specific (i.e., the difference of intra-subject and inter-subject$R^{2}$is significantly positive), with the notable exception of sub-03’s data which is modeled as well by other subjects’ models. At lagl=3, the subject specificity of the models is less noticeable, with more cases where the model is actually performing better on other subject’s data (i.e., the$R^{2}$difference being significantly negative). At lagl=6this trend is even more noticeable, with more of such cases. The subject inhomogeneity is also more marked at lagl=6, with a greater discrepancy in the$R^{2}$differences. Moreover the subject specificity of each model seems linked to its intra-subject performance (seeFig. 5a): the models with the highest$R^{2}$on their own subject are the one with the highest subject specificity. Thus, it appears that even though all models are better at modeling their own subject’s data at short lags, this specificity is less pronounced at longer lags.

**Fig. 5.**
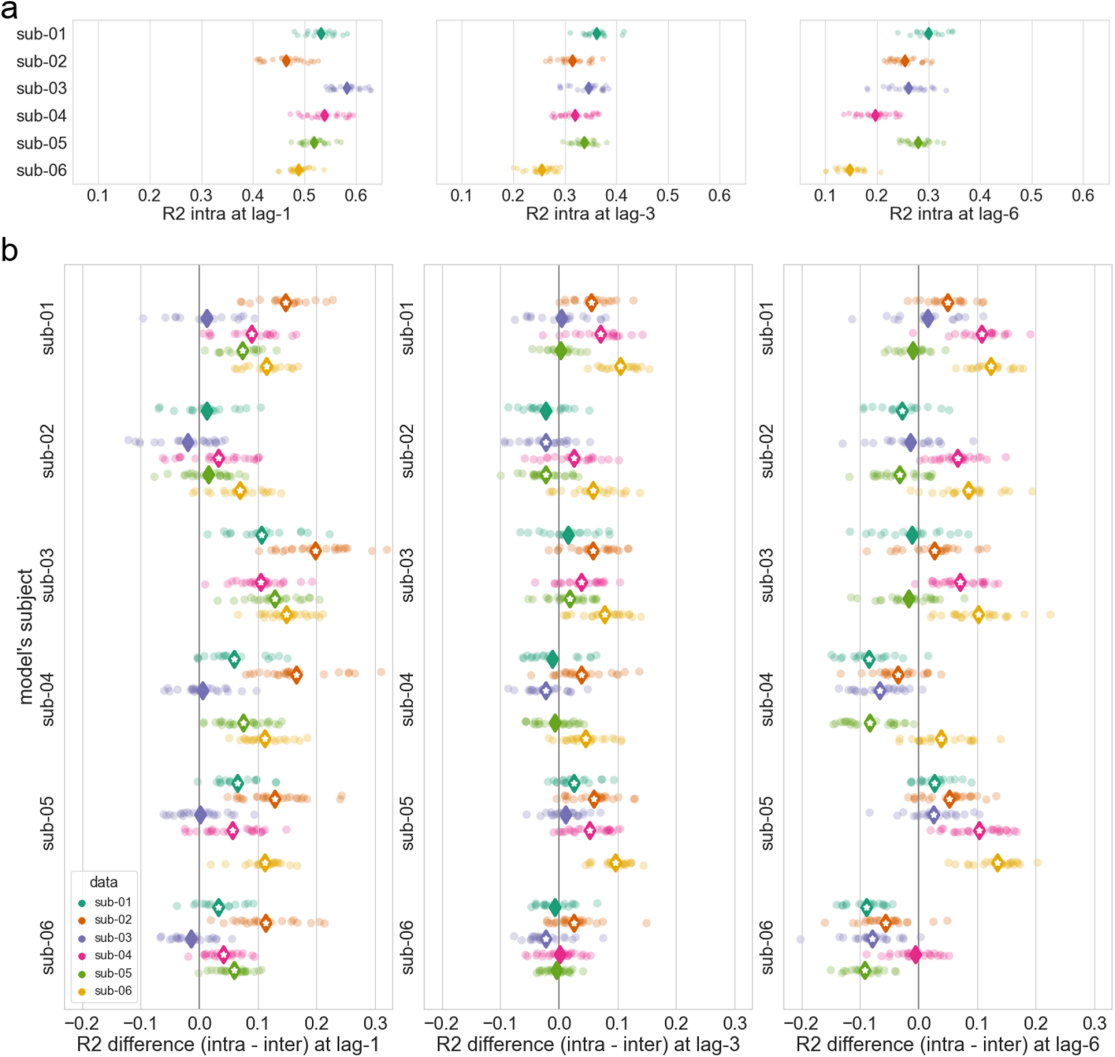
Subject specificity of the Chebnet models. (a)$R^{2}$in cortex for intra-subject and for lagsl=1,l=3andl=6TR. The$R^{2}$scores are averaged across parcels, each sample (dot) corresponds to an acquisition run, and the average across runs is denoted by a diamond. Intra-subject means that each model evaluated on data of the same subject it was trained on. (b) Difference of$R^{2}$in cortex between intra- and inter-subject, for lagsl=1,l=3andl=6TR. Each sample (dot) corresponds to an acquisition run and represents the difference between the$R^{2}$(averaged across parcels) of the model on its training subject for this run and the$R^{2}$on another subject for the same run. The mean difference across runs is denoted by a diamond, with a white star denoting the significance of a two-sided Wilcoxon test (p<0.05).

### 3.6 Group model perform worse than individual models

A model trained on the same amount of data but with the data coming from all of the 6 subjects performed significantly worse than the individual models. On average across runs and subjects, the effect size ranged between 0.03 and 0.055, and the difference increased with lag (except for lag 1 where the difference is bigger than for lag 2), seeFigure 6.

**Fig. 6.**
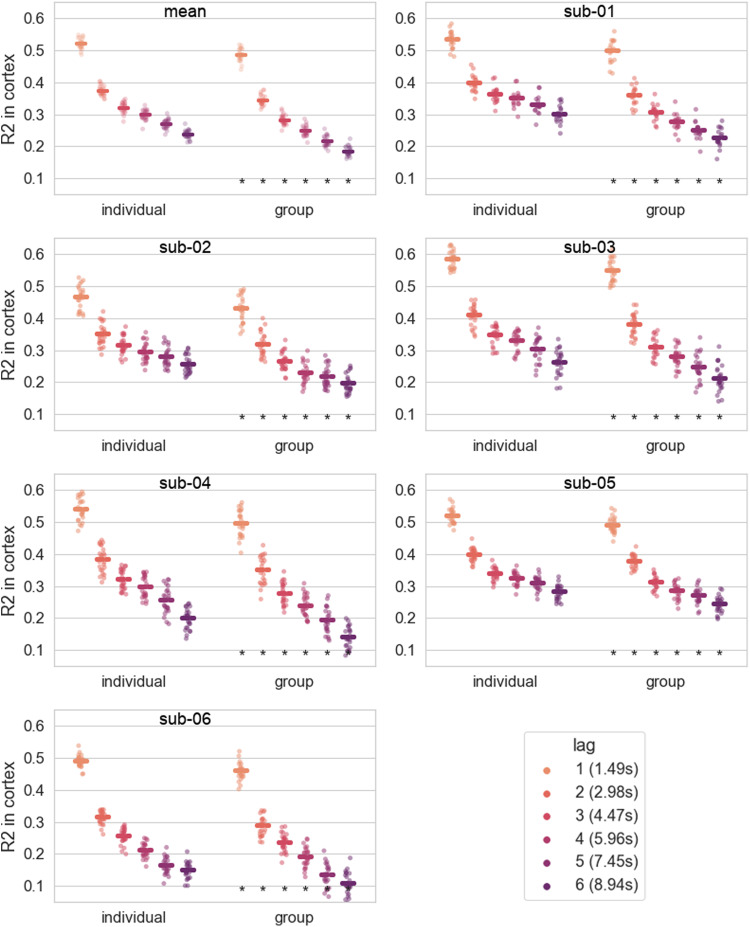
Comparison between the individual models and a group model trained on a similar amount of data.

### 3.7 The accuracy of auto-regression is highly dependent on lag (shorter is easier) and subjects

**Fig. 7.**
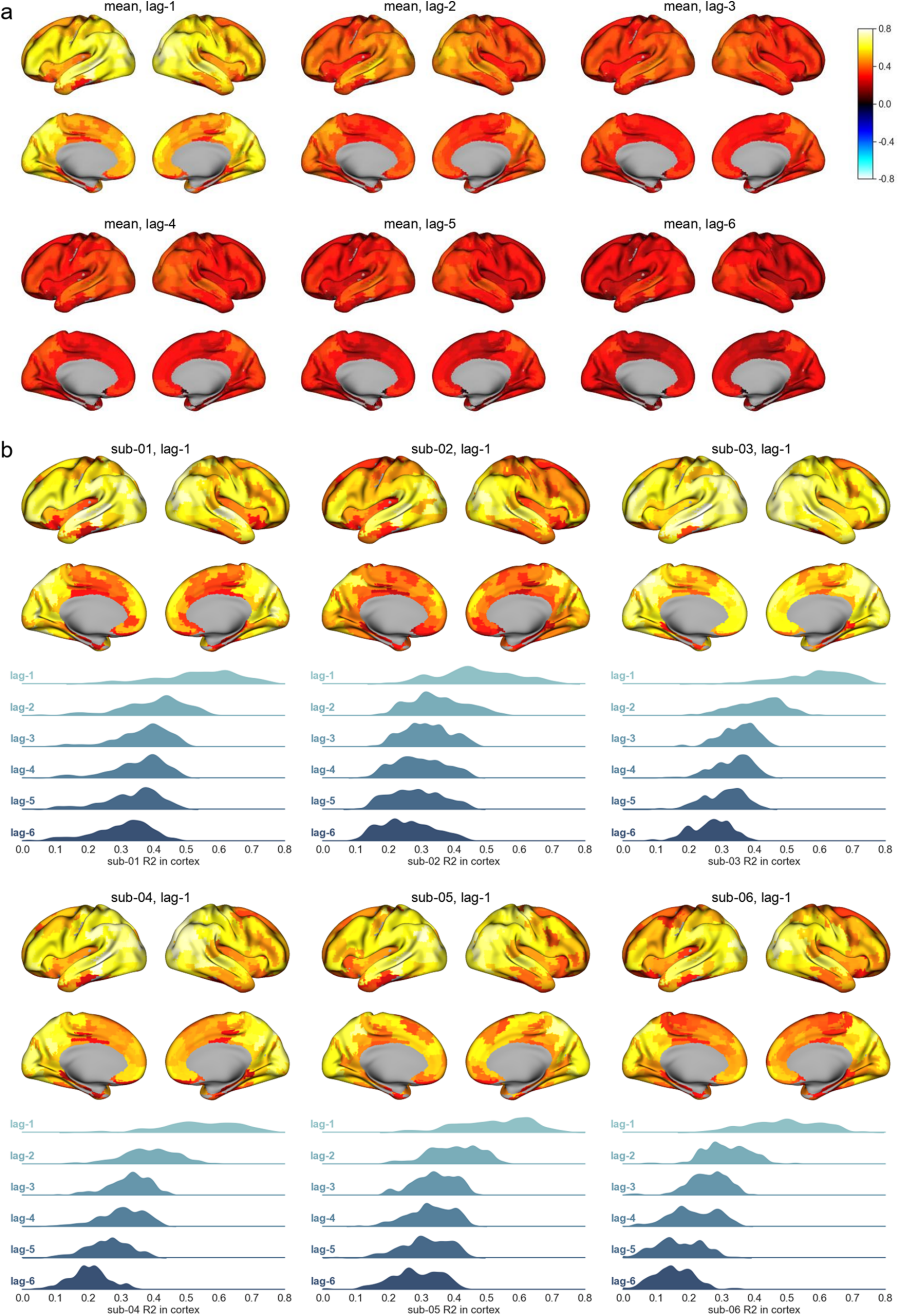
Distributions of$R^{2}$score of the Chebnets on test set. (a) Surface map of mean$R^{2}$over subject for lagl=1tol=6TRs. (b) For each subject surface map of mean$R^{2}$for lagl=1TR and distributions of$R^{2}$in cortex for lagsl=1tol=6TRs. The spatial distributions and distributions per lag are very consistent across subjects, and thus very consistent with the mean distribution.

Given the excellent performance of the Chebnets in our model comparison, we aimed to characterize more fully its modeling of brain activity. We first checked how the lag of time prediction (up to 8.94 s in the future) impacted the accuracy of the prediction across the brain. We specifically examined the spatial distribution of$R^{2}$across the 197 brain parcels, on average across subjects as well as separately for each subject (Fig. 7). The same trend was systematically observed across all subjects: the whole distribution of$R^{2}$shifted downwards with increasing lags, with high values (average range between 5th and 95th percentiles: 0.33 to 0.68 across subjects) for lagl=1, and low values for lagl=6(average range: 0.15 to 0.33 across subjects). The values of$R^{2}$also shifted systematically across subjects, with sub-03 showing the largest$R^{2}$(range 0.40–0.73 at lagl=1) and sub-06 the lowest$R^{2}$(range 0.32–0.66 at lagl=1). We thus found that the accuracy of auto-regression was highly dependent on the lag, with shorter lags being easier, and also highly subject-specific.

### 3.8 Most brain regions are accurately modeled at lag 1, and regions with low ${\text{R}}^{\text{2}}$ are consistent across subjects

We also checked the consistency of$R^{2}$brain maps across subjects, to see if some regions had systematic high (or low) auto-regression accuracy (Fig. 7b). We observed some heterogeneity in$R^{2}$values across the brains. Some regions consistently reached top$R^{2}$values (above .7) for all subjects, including some visual, temporal, and prefrontal regions. Others were consistently poorly predicted, with$R^{2}$around .5, such as ventral temporal and orbital cortices. These regions are known to suffer from signal distortions and low (or absent) SNR. The dorsal sensorimotor regions as well as mid-cingulate cortices also showed low$R^{2}$(around .5), but only in some subjects: the effect was much less pronounced in sub-03 in particular. Overall, we observed that Chebnets auto-regression at lagl=1worked well throughout the brain, with a few exceptions consistent across subjects.

### 3.9 At longer lags, the Chebnets mainly capture slow dynamics in the BOLD signal

**Fig. 8.**
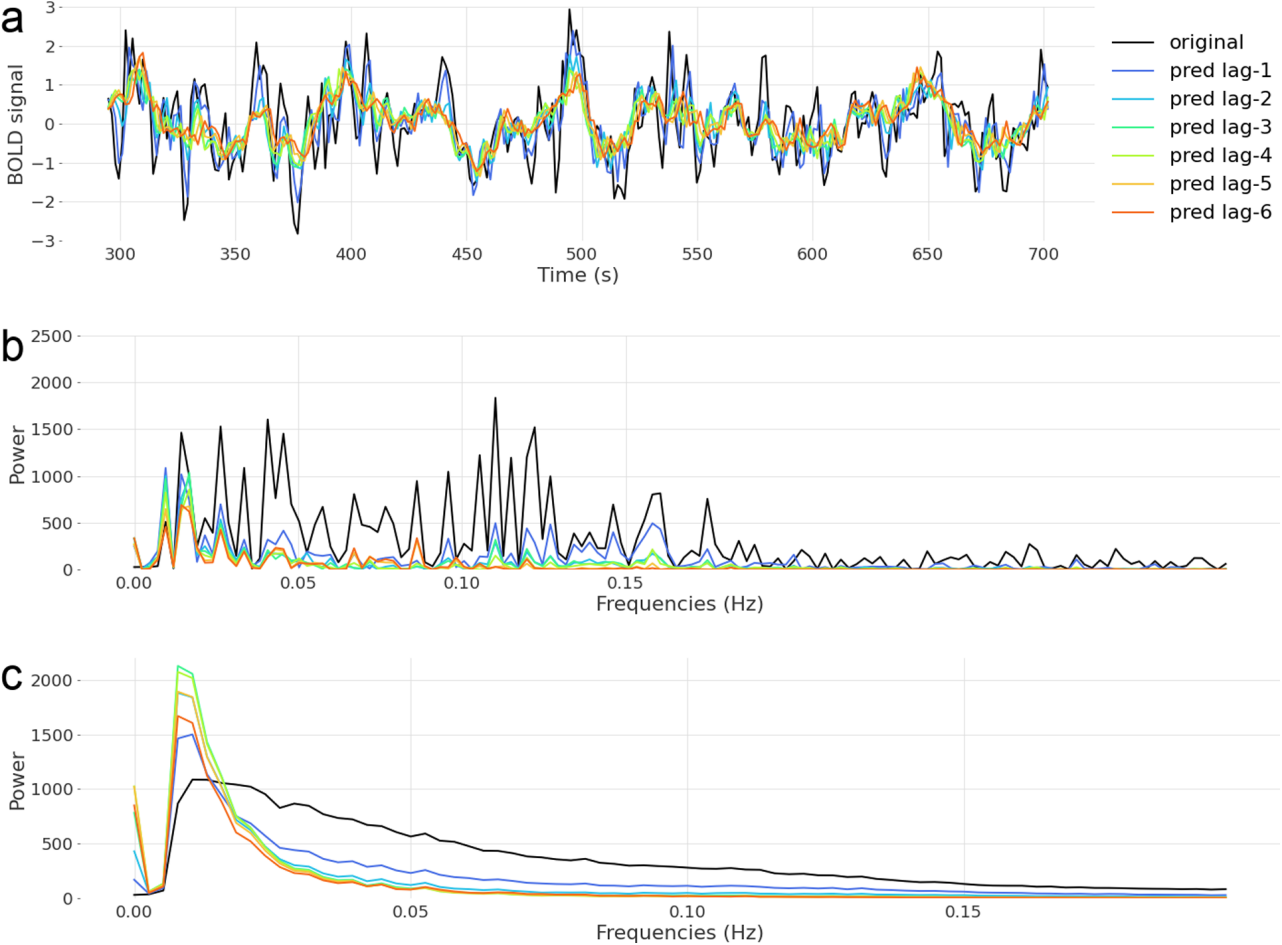
(a) Example of a time-series with Chebnet predictions at different lags. The data correspond to the recording of sub-01’s brain activity while watching the second half of the third episode of the second season of Friends, in a parcel in the occipital lobe (parcel 59 of MIST197). The longer the lag, the less the prediction captures the peaks, the more it corresponds to a smoothed version of the original signal. (b) Power spectra of the signals plotted in a. The longer the lags, the less the predicted signal has contributions in higher frequencies. This suggests that the long-term predictions capture mainly the low frequencies of the original signal. (c) Average power spectra for the original signal and predictions at each lag, averaged over subjects, runs, and parcels. It confirms that on average, the longer the lag, the more the spectrum is skewed towards low frequencies.

As BOLD fluctuations driven by neuronal activity are known to be dominated by ultra-slow frequencies, we looked at the power spectrum of the predicted signal at different lags for the Chebnets (seeFig. 8). The power spectra were computed with numpy’s implementation of the discrete Fast Fourier Transform (Cooley & Tukey, 1965). We found that predictions were indeed dominated by low frequencies. In addition, predicted dynamics over longer lags had even less power in the higher frequencies (above 0.025) than the predictions at shorter lags. This observation was visible on the spectra of a single parcel, run, and subject (Fig. 8b) as well as on the mean spectra, averaged across subjects, runs, and parcels (Fig. 8c). Chebnets predictions for longer lags captured mainly the slow dynamics of the signal.

### 3.10 The Chebnets generalize across a wide range of video stimuli as well as resting-state, and perform best on resting-state data at the longest lag

The training of the model as well as all previous evaluations on test data were done with the same class of stimuli: watching episodes of the TV series*friends*. We next aimed to quantify if the Chebnet autoregressive models would generalize to different tasks. We evaluated the Chebnets performance on resting-state data and data acquired during movie watching, testing a variety of genres (documentary, action, drama). As can be seen inFigure 9, the scores on movie watching tasks were very similar to*friends*, which was somewhat expected as all stimuli were variants of video watching. The Chebnet models generalized well on resting-state data too. For the longest prediction forecast (lagl=6), the best performance of Chebnets was consistently observed for resting-state data compared to video stimuli, including the test set of the*friends*dataset. We concluded that individual Chebnet auto-regressive models generalized well across a wide range of video stimuli, and were even better on resting-state data at long lags.

**Fig. 9.**
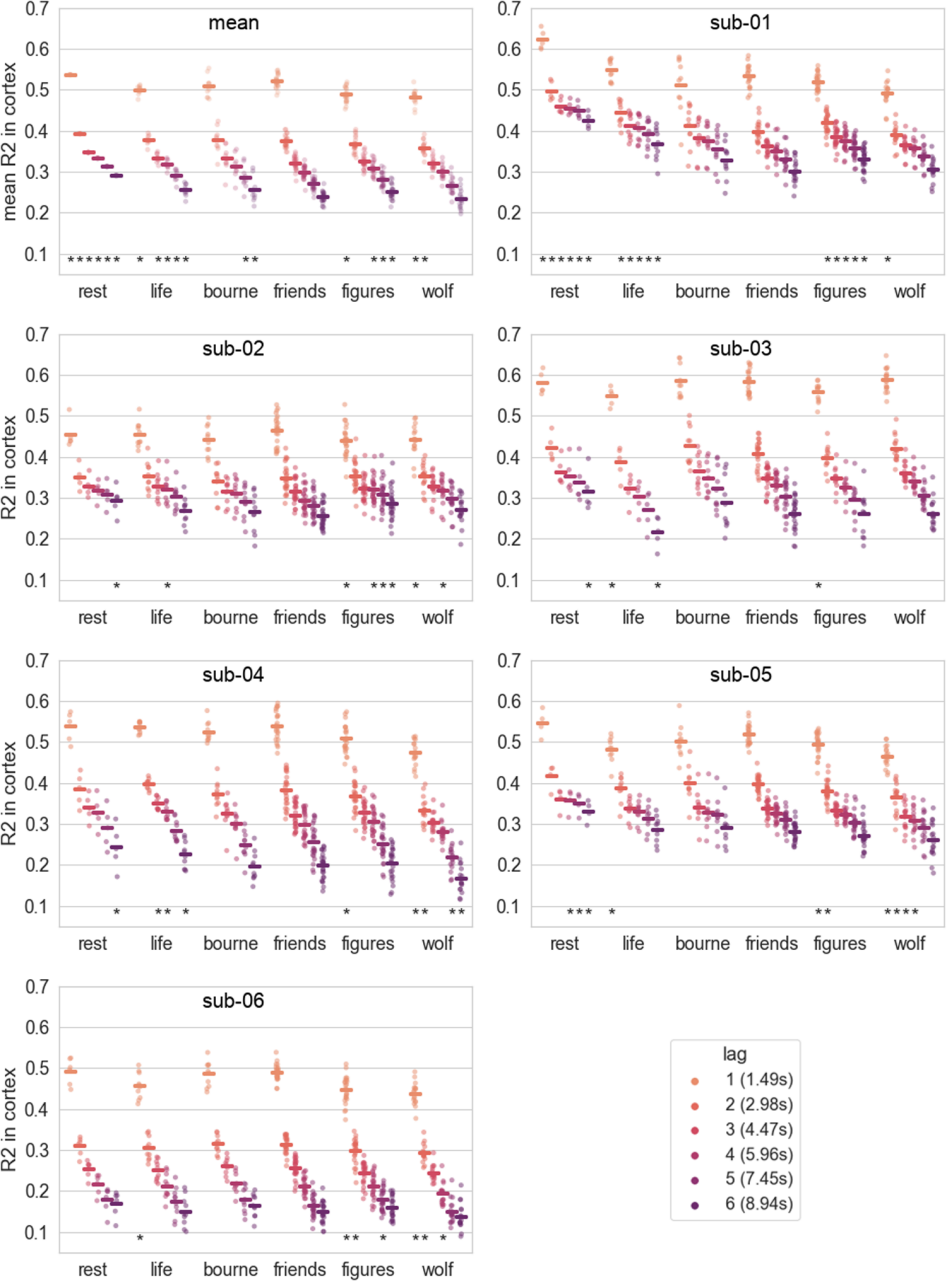
Task generalization of the Chebnet models. For the mean across subjects (top left) and each subject, the mean$R^{2}$in cortex is plotted for different tasks and each lag.*friends*corresponds to the test set.*rest*corresponds to 5 runs of 15 min of resting state for each participant.*bourne*,*figures*,*life*, and*wolf*correspond to the watching of the movies*Bourne Supremacy, Hidden Figures, Life*, and*The Wolf of Wall Street*respectively. For each movie task, the model appears to generalize well, which is not surprising as movie watching is a very similar task to watching a TV show. The models generalize well on resting-state data and it appears that the Chebnets perform better for longer lags on resting-state data. This is consistent with the idea that on longer lags the model captures slow dynamics, as it is known that resting-state data are dominated by slow dynamics (Biswal et al., 1995;Cordes et al., 2001). The asterisks denote a significant difference between the$R^{2}$scores of a task and the*friends*task, for a given subject and prediction lag (p<0.05for a two-sided Wilcoxon test).

### 3.11 Regressing out the shared response degrades the Chebnets accuracy for all lags

We compared the performance of the Chebnets to other Chebnets trained with the same hyper-parameters but on data where the shared response is regressed out. The difference of performance between the models trained and evaluated on data with and without the shared response appears statistically significant for almost all subjects and prediction lags, as can be seen inFigure 10. The models trained without the shared response explain less variance, but the drops in$R^{2}$appear moderate compared to the differences between model architectures or subjects, representing a 5 to 15% drop of the original scores (range 0.05 and 0.02 difference in$R^{2}$).

**Fig. 10.**
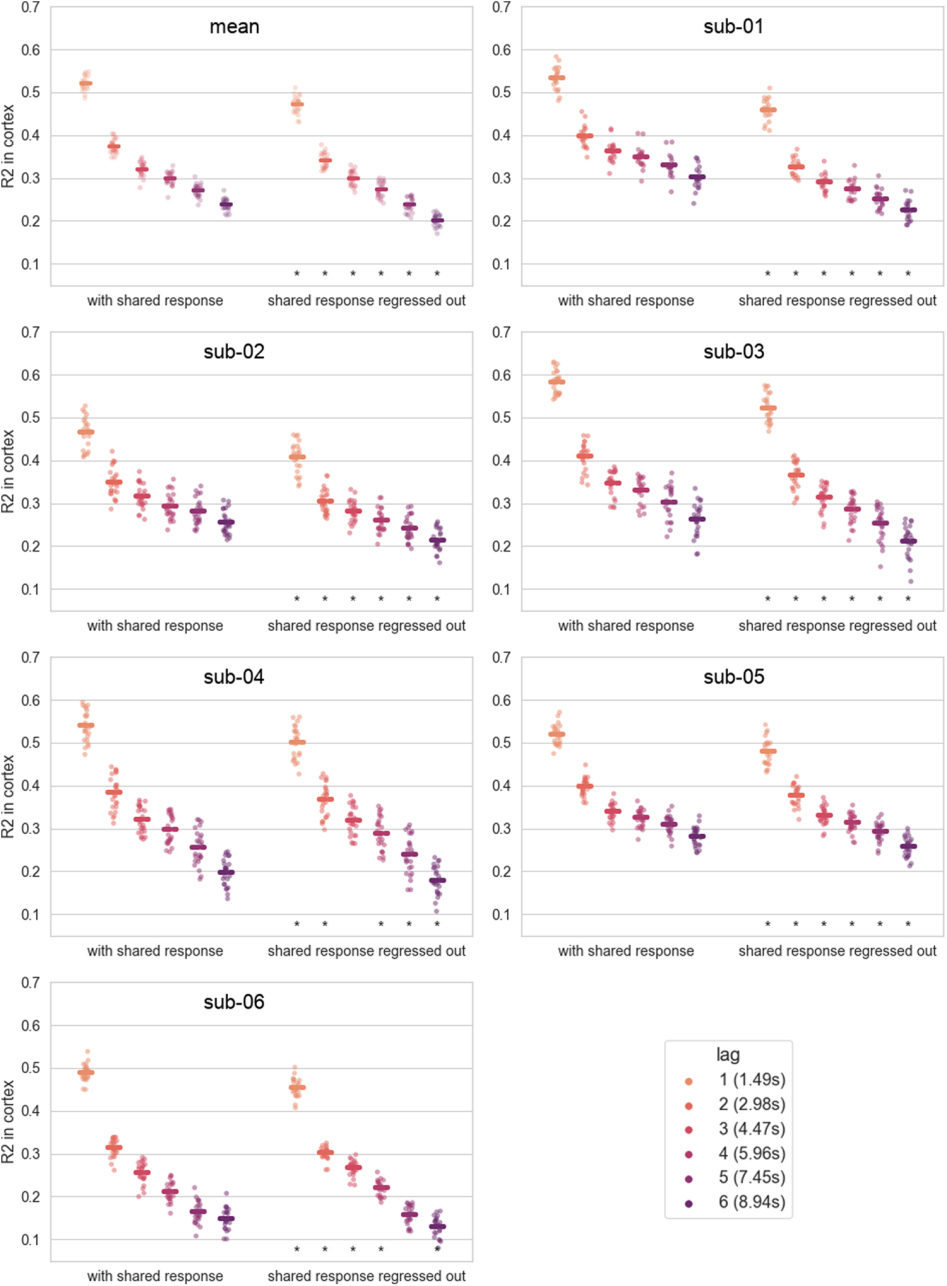
Comparison between Chebnets trained on the original BOLD signal and Chebnets trained on BOLD signal with the shared response repressed out. The asterisks denote a significant difference between the$R^{2}$scores of both types of models for one lag. The significance corresponds to a p-value ¡ 0.05 for a two-sided Wilcoxon test.

### 3.12 Seed-based connectivity maps suggest that the predicted slow dynamics relate to brain network organization

The Chebnets’ predictions for longer lags correspond to lower frequency dynamics, as shown inFigure 8. Slow dynamics in fMRI BOLD signals are a hallmark of brain connectivity at rest (Biswal et al., 1995;Cordes et al., 2001), yet may also reflect various noise sources in particular motion artifacts (Scheel et al., 2014). We wanted to verify if the slow dynamics modeled by the Chebnets were capturing artifacts or meaningful signals. In order to do this, we computed the seed-based connectivity maps of the original signal and prediction at different lags, with seeds in 6 of the 7 Yeo networks (Thomas Yeo et al., 2011). We excluded the seventh network because it is composed of regions with high signal loss and distortions due to field inhomogeneity. The maps for the DMN seed, for each subject and for the original signal and predictions at lagsl=1,l=3andl=6, are presented inFigure 11. The maps were thresholded to only show the top 10% most correlated voxels. For each subject, the maps of all lags are very spatially consistent with the map from the original signal, which correspond to the expected DMN network. The levels of connectivity decreased with increasing lags, as expected since at longer lags less variance of the original signal is explained. The same observation holds for all other networks; see section II of theSupplementary Material. The consistency of these maps suggests that the modeled signal corresponds to the major intrinsic connectivity networks which are robustly found in task conditions (Gordon et al., 2017), and not to motion artifacts.

**Fig. 11.**
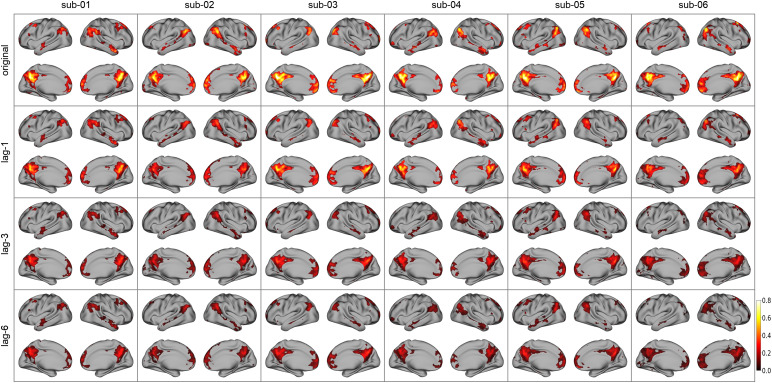
Seed-based connectivity maps with a seed in DMN (precuneus). For each subject, the correlation between the signal in every voxel and the signal in the region containing the seed of MNI coordinates (-7, -52, 26) is computed and presented on the first row (“original”). On following rows, the same correlation computation is done, but replacing the signal in the DMN region with the predicted signal in this region, predicted at lagl=1,l=3, andl=6for the second, third, and fourth row, respectively. Each map is thresholded to only show the 10% of voxels with the highest values. We can see that the levels of correlation diminish with increasing lag, the regions with maximum correlation stay consistent, and correspond to the DMN. This suggests that even the slow dynamics predicted at lagl=6correspond to brain dynamics and not slow drifts caused by movement artifacts. If it were the case, we would expect to see artifacts in the correlation maps, in particular higher correlations in peripheral regions. Similar maps for seeds in other networks can be seen in section II of theSupplementary Material.

## 4 Discussion

### 4.1 Summary

This study is a benchmark of auto-regressive models of fMRI time-series, using a massive deep individual dataset. We compared different model architectures trained with fMRI data collected while participants watched episodes from the Friends TV series. Only the Chebnets could outperform the linear models across all prediction lags, with linear Chebnets outperforming the standard non-linear ones. The Chebnet and univariate (linear and MLP) models had a clear advantage for predictions at longer lags (l=3tol=6TRs), while at short lags (l=1) Chebnets and multivariate linear models performed best. Chebnets scores scaled with the amount of training data (up to 9 h), following an increasing trend with no clear saturation of performance. The Chebnet models were also found to be significantly subject specific at lagl=1, but this subject specificity decreased with increasing lags. Chebnet models had good performance across parcels in the cortex, for all time lags, with lower$R^{2}$prediction localized in regions of low SNR, consistent across subjects. The time-series predicted by Chebnets were dominated by slow fluctuations, especially for predictions at long time lag, and generalized well to different video stimuli and resting-state data. Using time-series generated by Chebnets to generate seed-based connectivity maps in the canonical resting-state networks, it was apparent that connectivity maps based on Chebnet predictions were consistent with traditional functional connectivity maps. Taken together, these findings demonstrate that Chebnets capture ongoing slow brain fluctuations underlying common functional connectivity analyses.

### 4.2 Model comparison

#### 4.2.1 Key features of high performance models

In our initial model comparison, except for the Chebnet models, we found the linear models to outperform the non-linear ones. This observation indicates that the use of non-linearities does not appear as a significant advantage to model the BOLD time-series, at least for our data regime. Additionally, a sensitivity analysis on the non-linear activation functions of the Chebnets revealed that linear Chenets outperform non-linear ones. This result came as a surprise, as we expected models with non-linearities to benefit from higher expressivity. Therefore, the overall best model identified by this study is a linear Chebnet. Although we expect the analysis done on the non-linear Chebnet to hold similar results with linear Chebnets, additional work on the data scaling, subject specificity, and task generalization of the linear Chebnet would be required to fully rule out any benefit from the non-linearities.

The lack of a clear advantage of the non-linear models can be, in part, explained by the fact that the measured BOLD time-series appeared to be stationary. Augmented Dickey-Fuller and Kwiatkowski-Phillips-Schmidt-Shin tests for stationarity were performed on the time-series of the test set, revealing no significant evidence of non-stationarity.

The superiority of the multivariate models over the univariate ones at short lags (l=1andl=2TRs) indicates that the modeling of spatial autocorrelation plays a key role to model the faster BOLD dynamics. This interpretation is also supported by the results of the graph comparison analysis, which showed that the functional connectivity graph provides a significant improvement over random or spatial graphs only at short lags.

On the contrary, univariate models clearly outperform multivariate models at longer lags (l=3to 6 TRs). Since they do not model spatial interactions, the linear models have a much lower parameter complexity and thus can scale to much longer input sequences of past time points (kinequation (1)). The best univariate methods (linear and MLP) had respectively input length ofk=256(6 min 21 s) and128(3 min 10 s). Whereas for other models the optimal input lengths were much shorter, ranging fromk=1TRs (1.49 s) tok=64TRs (1 min 35 s). We hypothesize that good prediction scores for long time lags reflect this use of longer input lengths.

The Chebnet models benefit from a pre-computed functional connectivity graph, which acts as a powerful prior for modeling spatial autocorrelation of the brain dynamics. The use of graph convolutions allows for modeling spatial interactions at low parameter complexity, enabling the use of long input sequences (k=192, 4 min 46 s) which is reflected in the Chebnets’ training time. The Chebnets can thus benefit from both the advantages of the multivariate and univariate models and outperform these models at short and long lags. The superior performance of graph convolutional networks over VAR to model BOLD time-series is consistent with Wein’s group studies (Wein et al., 2021,^2022^). Our results extend these findings to the individual level. It should be noted that the longer input sequences render the Chebnet and univariate models inapplicable to short time-series. In the case of short time-series, linear multivariate might therefore be preferable.

While the other multivariate non-linear model architectures presented in this study could not scale well with long input sequences with our amount of training data, it is possible that using larger amounts of data could enable them to better scale with the input length, and thus perform better for predictions at longer lags. Additionally, comparing the models through other criteria than raw accuracy, such as model interpretability, would be an equally interesting direction and could provide a different ranking of the model architectures. We note that the recent study byThomas et al. (2023)have found that a transformer-decoder architecture could be successfully trained at the group level for short-term auto-regression and mental state decoding using a massive collection of datasets.

Our selection of model architectures did not include regular CNNs, as they require the data to be structured on a grid. We used fMRI data projected on an atlas, which does not have a grid structure. Applications of regular CNNs on functional connectomes have relied on a somewhat arbitrary order of connections (Leming & Suckling, 2021), therefore we found graph convolutions to be better suited for our type of data. Recent works have shown that the combination of sel-attention with regular convolutions can improve the capacity of the models (Dai et al., 2021), with such hybrid models being successfully used on brain imaging data (Tao et al., 2023;Y. Wu et al., 2019). Therefore, we note that a variant of the graph convolutions combined with self-attention constitutes a promising path to improve the Chebnet.

It should be noted that the input length values tried in the hyper-parameter grid search vary from a model to another because of the difference in the computational needs of each model. A more systematic comparison with the exact same input length values for each model would be interesting, but it was out of reach given our computational resources (e.g., the gridsearch for the Temporal Fusion Transformer model took about 4 GPU days). Moreover, the hyper-parameter search could be replaced by more recent automated hyper-parameter tuning approaches, such as Population Based Training (Parker-Holder et al., 2021), Hyperband (Li et al., 2018), or Bayesian Optimisation (Joy et al., 2016). An automated approach would remove the bias in the choices of values in each grid search.

#### 4.2.2 Difference in prediction method between hyper-parameter search and subsequent performance evaluations

During the hyper-parameter search, we trained a separate Chebnet model for each prediction lag and subject. This approach involved training six different models in parallel, where each model predicted the values for time stepst+1tot+6based on a window of past time steps fromt−ktot. We refer to this method as the multi-step prediction. To determine the best hyper-parameters, we selected the ones with the highest validation$R^{2}$score, which was averaged over subjects and lags. When comparing the models and reporting$R^{2}$values in the results sections, we obtained predictions using only the models to predict thet+1time step. We then recursively fed back these predictions to the model to further predict the time stepst+2tot+6. This prediction approach is referred to as the iterated single-step prediction method. We opted to use different prediction methods for evaluation because recurrent models can only be used with the iterated single-step method. By employing this method for all models, we ensured a more fair comparison. Moreover, we observed that the differences in performance between the two prediction methods were small compared to the differences in performance between models, tasks, or subjects; see Figure VI inSupplementary Material.

### 4.3 Data scaling

Large data are beneficial for auto-regressive models, and this study did not reach a ceiling. The large fMRI sample available for this study motivated the use of deep learning models, rather than only linear models. Deep learning models are, indeed, known to substantially improve their performance in high data regimes, for example, for natural image categorization (Barz & Denzler, 2020;Brigato & Iocchi, 2020). To verify that the use of larger amounts of data is beneficial to the Chebnets, we looked at performance scaling with increasing amounts of training data. We observed an exponential-like relationship, with fast increments in performance up until 3 h of training data, followed by a slower increase. The performance scaling curves did not appear to have reached saturation even with the maximal amount of training data available (9 h per subject). The increase in performance was more pronounced at short lags. Much smaller amounts of data (i.e., 30 min) have been shown to produce reliable individual estimates of static (zero-lag) functional connectivity (Gordon et al., 2017). Our results show that dynamic, space-time models require substantially more data and this observation extended to linear models as well. The data requirements to train an individual space-time brain encoding model using a deep convolutional neural network in vision experiments are more in line with our results: 12 h for saturating performance (Seeliger et al., 2021).

### 4.4 Subject specificity

This AR benchmark is a pure individual study based on a massive deep fMRI dataset. By contrast, most BOLD AR models are trained at a group level. Such group models have a lower noise ceiling than individual models due to inter-subject variability. To verify that the Chebnet models could take advantage of the individual modeling and capture parts of the inter-individual variance, we looked at the subject specificity of each model. For lag l = 1 TR, the models tended to perform significantly better on their own subject’s data rather than other subjects’ data, yet this effect reduced with longer lags and eventually disappeared at lag l = 6 TRs for some subjects. A model built by combining data from all individuals also performed worse than individual-specific models. Our models captured some individual-specific features, at least for short lags. Individual-specific features were recently found to dominate functional networks at rest (Gratton et al., 2018). Individual brain decoding models have also been shown to learn subject-specific features (Porter et al., 2023). Our results are not directly comparable to those of Wein and colleagues, who used group models with a band-pass filter (0.04–0.07 Hz) to suppress the high frequencies of the BOLD signal, while we only used a high-pass filter (>0.01 Hz). In our results, subject specificity is stronger at short lags which capture higher frequencies. The subject specificity is less apparent for longer lags where only very slow dynamics are modeled. We speculate that group models may capture only slow dynamics, whereas individual models capture a wider range of frequencies. The advantage of individual models over group ones might not remain true with increasing amounts of data, as a significantly larger training dataset would enable the use of larger models which could potentially capture the individual specificities better. We did not expect marked differences in hyper-parameters across individuals and therefore optimized the hyper-parameters for all subjects. Optimizing the hyper-parameters independently for each subject could potentially increase the subject-specificity of the individual models.

In most cases, the models of other subjects perform equally well on sub-03’s data as they do on their own data, while sub-03’s model demonstrates the best intra-subject performance. We hypothesize that sub-03 potentially exhibits a higher signal-to-noise ratio (SNR) in regions predominantly influenced by evoked activity, which could be attributed to the subject having the least amount of motion. Our observations indicate that incorporating evoked activity yields a favorable impact on prediction accuracy. Therefore, it is plausible that the models of other subjects compensate for their subject-specific characteristics when applied to sub-03’s data by accurately predicting the shared response.

### 4.5 Spatiotemporal structures

#### 4.5.1 Frequency content and seed-based connectivity

Our results show that Chebnets AR modeling of BOLD signals at long lags rely on slow temporal fluctuations, as the power spectrums of the predicted signals were dominated by low frequencies (0.01–0.02 Hz). This is consistent with the prominence of slow fluctuations in spontaneous brain activity at rest (Biswal et al., 1995;Cordes et al., 2001). In addition, seed-based connectivity maps between original BOLD signal and a seed-based on predicted signals at different lags converged towards canonical patterns of co-fluctuations highly consistent with established brain networks extensively described during rest (Thomas Yeo et al., 2011). Taken together, these findings suggest that auto-regressive models do capture physiological fluctuations linked to neuronal activity in distributed brain networks, likely of similar origins to those giving rise to functional connectivity measures in fMRI.

#### 4.5.2 Task generalization

We wanted to check whether the Chebnet AR models learned spatiotemporal patterns specific to the cognitive context of the training data, that is, the Friends TV series. The AR models appeared to generalize well to various other types of video stimuli, an heterogeneous series of movies including both action, drama, and life documentary. The AR models also generalized well on resting-state data, and actually tended to perform better for longer lags (l=3tol=6TRs). This good generalizability to resting-state data suggests that the AR models’ performance is mainly driven by the actual neural dynamics rather than confounding factors derived from the narrative structure of the stimulus, in particular at longer lags. Looking at the generalizability of the Chebnets to more various stimuli and types of task would be an interesting complement to these results, as it has been shown that the functional state of the brain is task-dependent (Gratton et al., 2018;Shine & Poldrack, 2018), and that the changes in functional state according to the tasks are subject-specific (Porter et al., 2023). In addition, task states can also impact functional brain parcellations (Salehi et al., 2020), and our use of a single parcellation represents a limitation. It would be interesting to replicate our model comparison with a more flexible spatial model able to capture reconfigurations of parcels for different tasks. We speculate that the models would not generalize well to finer spatiotemporal resolutions, because of the increase in complexity entailed by higher dimensional data, and the domination of slow dynamics would not leverage higher sampling frequencies. New releases of CNeuromod data will make it possible to test the generalizability to other task modalities in future work.

#### 4.5.3 Evoked response and intrinsic activity

Auto-regressive models are often applied on the residuals of a general linear model, or resting-state activity. In this work, we used instead data collected while subjects watched a TV show. We selected this experimental paradigm because it was engaging for participants while collecting a massive amount of data in the scanner, while resting state is known to be prone to attention lapses and sleep (Tagliazucchi & Laufs, 2014). The brain activity can be divided into two components: the evoked response which results from the exposure to the stimulus and the intrinsic activity which reflects the ongoing fluctuations of the cognitive state which are independent of the stimuli. Some of the stimulus-evoked activity is shared across subjects, and the rest is subject-specific (idiosyncratic) (Nastase et al., 2019). All these components reflect the same type of neuronal activity coupled with hemodynamics, and auto-regressive models should in theory be applicable to any of these components, or their combination. We investigated how auto-regressive models performed after regressing out the shared response across subjects, mimicking residuals of a general linear model in task-based fMRI.

The Chebnets trained and evaluated on the estimated intrinsic activity performed significantly worse than the ones trained and evaluated on the whole BOLD signal, although the difference in performance was small. These results seem to indicate that the Chebnets are adequate to capture substantial variance in the shared response, and also capture variance in idiosyncratic as well as spontaneous activity. This was further supported by the excellent generalization of the models to resting-state data.

### 4.6 Implication

Multivariate auto-regressive models (MAR) model the spatiotemporal interactions in a multivariate time-series, and have long been proposed as a method to interpret functional interactions between brain regions. Specifically, the parameters of a trained MAR model can be interpreted as indicators of cross-region interactions, notably using Granger causality (Harrison et al., 2003;Rogers et al., 2010;Valdés-Sosa et al., 2005). We speculate that the higher predictability of graph convolutional networks (GCN) AR models makes them good candidates for methods based on ratios of explained variance to uncover functional interactions, such as the Granger causality. Although higher predictability does not guarantee better explainability, models with higher predictability capture larger amounts of the signal’s variance, which might make them more likely to capture some variance related to interactions explainable through subsequent methods like the Granger Causality.

Another possible application of Chebnet AR models is re-defining noise ceiling measurements for brain encoding experiments. The common methodology in brain encoding studies defines all signal variance that cannot be explained by the stimuli as noise, as measured through independent repeated presentations of the stimulus (Schrimpf et al., 2018). This definition intrinsically implies that any trial-to-trial variability in brain activity is noise, which cannot possibly be explained by a computational model. However, the subject specificity of our auto-regressive models shows that there is a part of the variance that is subject-specific and that can be modeled independently from the stimuli. Thus, taking into account predictable and subject specific slow dynamics could raise the noise ceiling. Better models of intrinsic brain dynamics may also help brain encoding models to capture interactions between intrinsic and evoked brain signals.

## 5 Conclusion

In this study, we compared several linear and deep models for the individual auto-regression of BOLD data during video watching. The Chebnet, a GCN, did appear to be better suited to model individual fMRI data. This result extends the similar conclusion of (Wein et al., 2021,^2022^) on BOLD auto-regression at the group level to the individual level. We interpret the GCN’s improvement over linear models to be mainly attributable to better complexity efficiency and balance between spatial and temporal interactions on long time ranges. This interpretation led us to test a linear version of the Chebnet architecture, which surprisingly outperformed the non-linear Chebnet. The signals generated by AR Chebnets were dominated by slow dynamics, and led to functionally relevant connectivity patterns. The AR Chebnets generalized well to movies of various genres and to resting-state data, especially at long prediction lags, for the latter. The models appeared to be subject-specific, especially at short lags, justifying the use of individual models and deep fMRI samples. The Chebnets improved their accuracy with increasing amounts of training data, with diminishing returns but no ceiling in performance with 9 h of data per subject.

AR models are a core element of many modeling tools of BOLD data, such as Granger Causality, and can also be used to generate synthetic brain data. Although better predictability does not always imply better explainability, better individual AR models could have a beneficial impact on the understanding of functional brain connectivity in future works. Explaining larger portions of variance in BOLD signals through auto-regression could also reshape the definition of noise ceiling in brain encoding experiments. Finally, our study presents a public dataset which can be used as a benchmark by the field to evaluate AR models with regards to their accuracy, generalizability, subject specificity, and data scaling.

## Supplementary Material

Supplementary Material

## Data Availability

The code for the experiments is publicly available at:https://github.com/FrancoisPgm/fmri-autoreg. The Neuromod datasets are available through an inter-institutional data transfer agreement. A complete description of the process to access the datasets is available at the following url:https://docs.cneuromod.ca/en/latest/ACCESS.html.
