# Supplementary material for: A benchmark of individual auto-regressive models in a massive fMRI dataset
Source: Imaging Neurosci (Camb). 2024 Jul 15;2:imag-2-00228. doi: 10.1162/imag_a_00228 (PMC12272179; doi:10.1162/imag_a_00228)
Supplement: Supplementary Material [file imag_a_00228-supp.pdf]

Supplementary material for “A benchmark of individual auto-regressive models in a massive fMRI dataset”

François Paugam[abc]*, Basile Pinsard[b], Guillaume Lajoie[ac], Pierre Bellec[ab]

a Université de Montréal, Montréal, Canada
b Centre de Recherche de l’Institut Universitaire de Gériatrie de Montréal, Montréal, Canada
c Mila - Institut Québécois d’Intelligence Artificielle, Montréal, Canada
* Corresponding author : francois.paugam@umontreal.ca

I: Examples of time-series and power spectrums for a run.

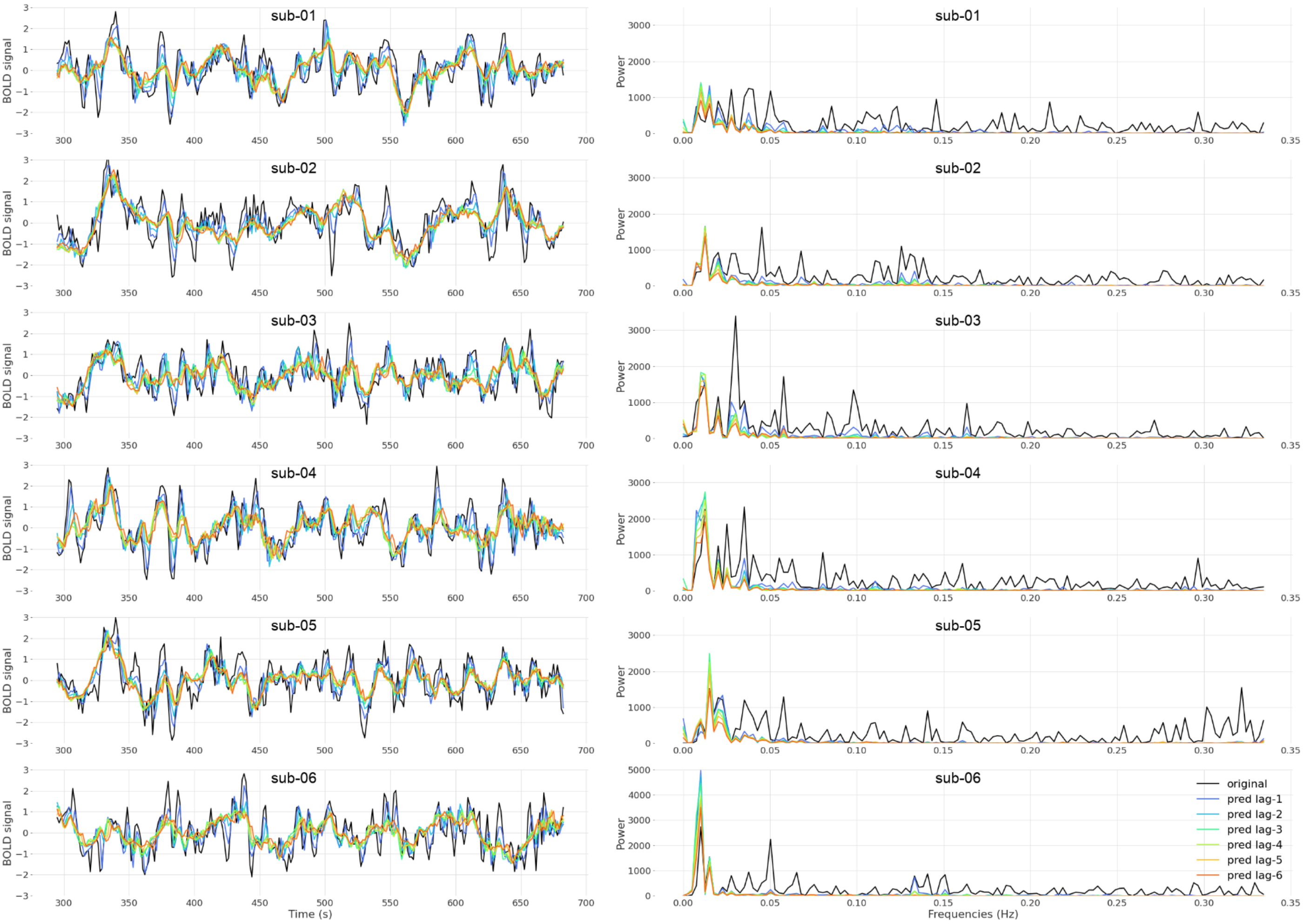

Fig I.1: Examples of the original (black) and predicted (colored) BOLD time-series in time (left) and frequency (right) spaces for one run for each subject.

II: Seed-based connectivity maps

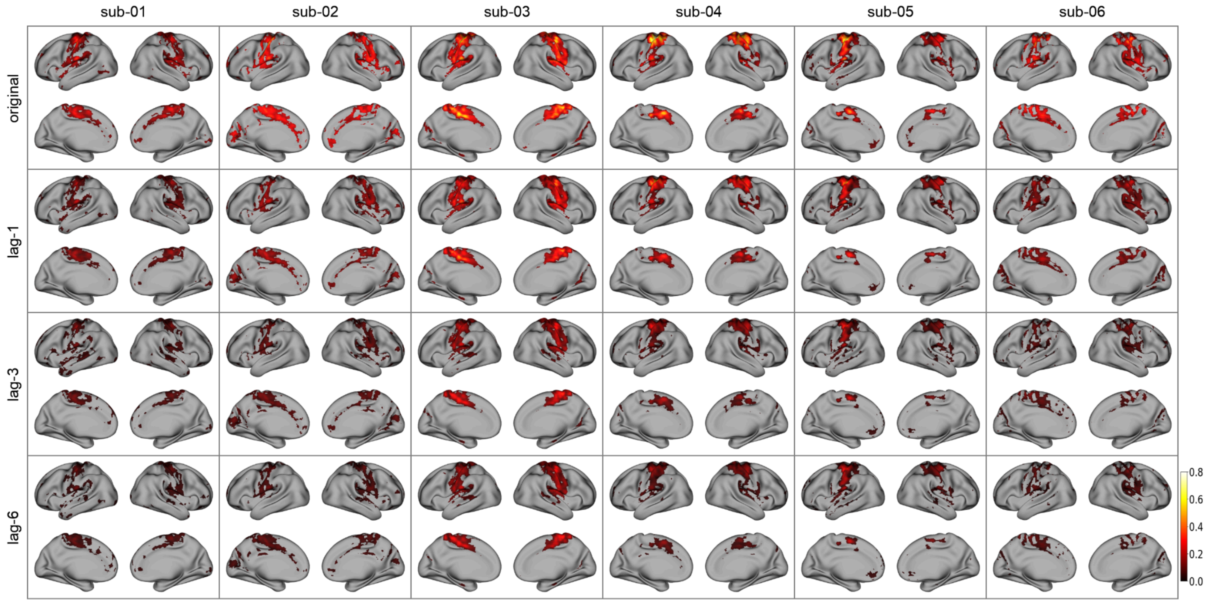

Fig II.1: Seed-based connectivity maps with a seed in the sensorimotor network (MNI coordinates -41, -20, 62).

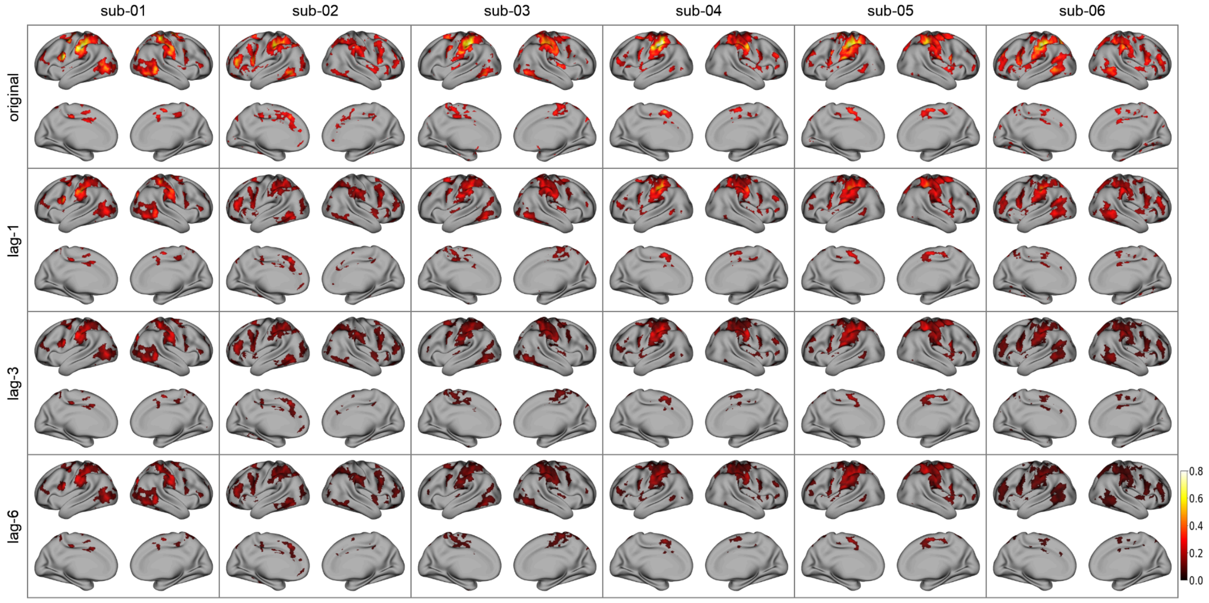

Fig II.2: Seed-based connectivity maps with a seed in the dorsal attentional network (MNI coordinates -34, -38, 44).

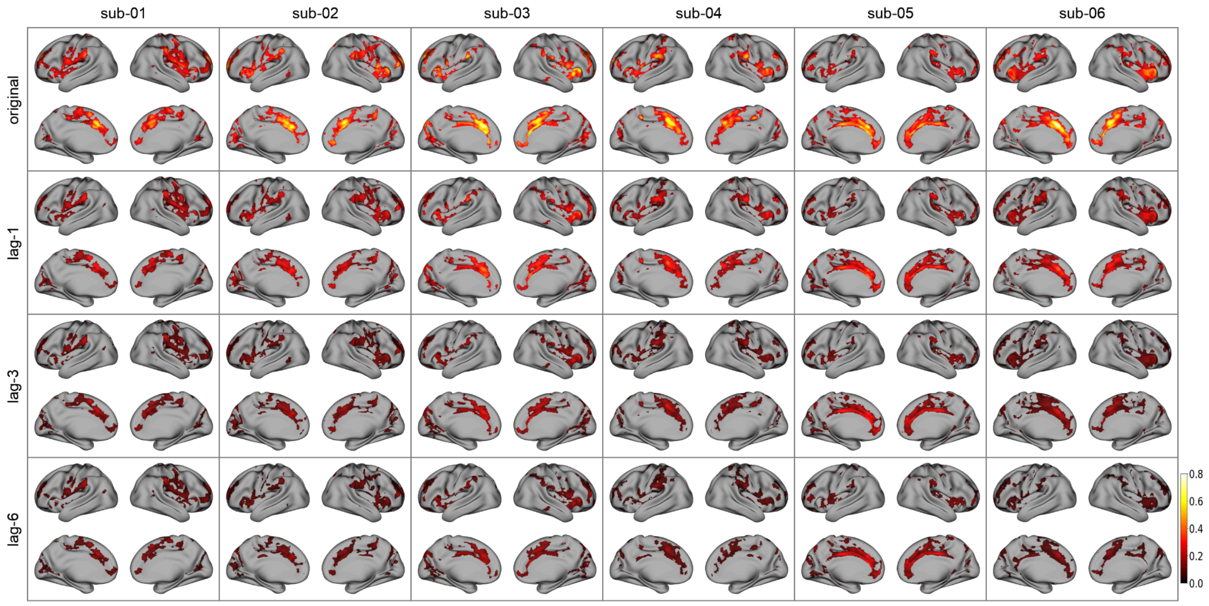

Fig II.3: Seed-based connectivity maps with a seed in the ventral attentional network (MNI coordinates -5, 15, 32).

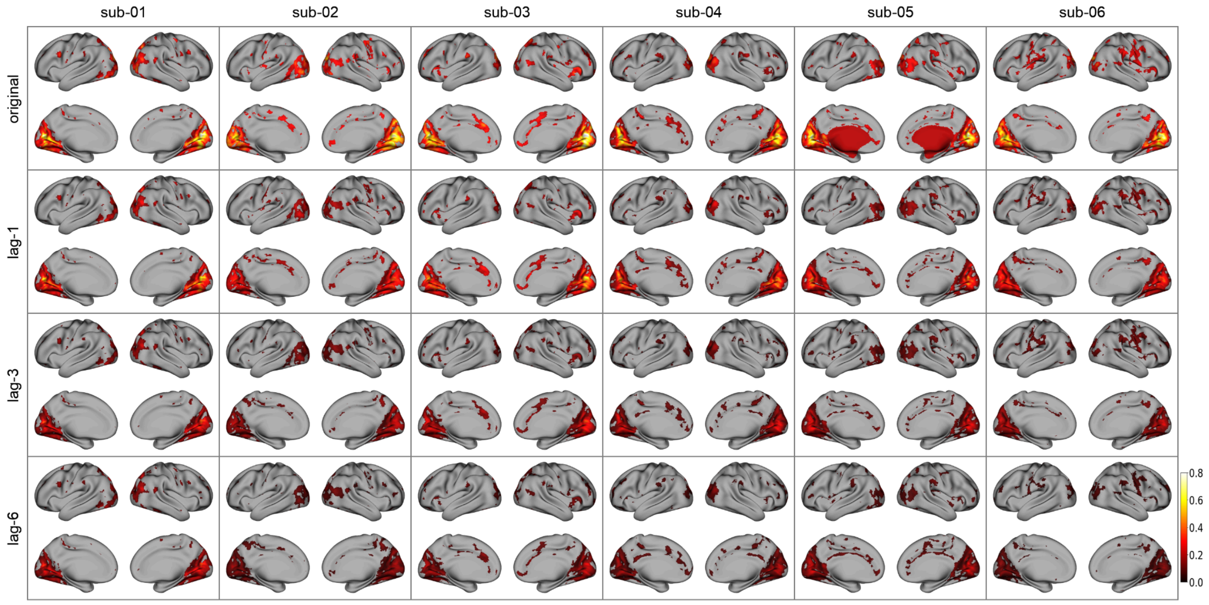

Fig II.4: Seed-based connectivity maps with a seed in the visual network (MNI coordinates -16, -74, 7).

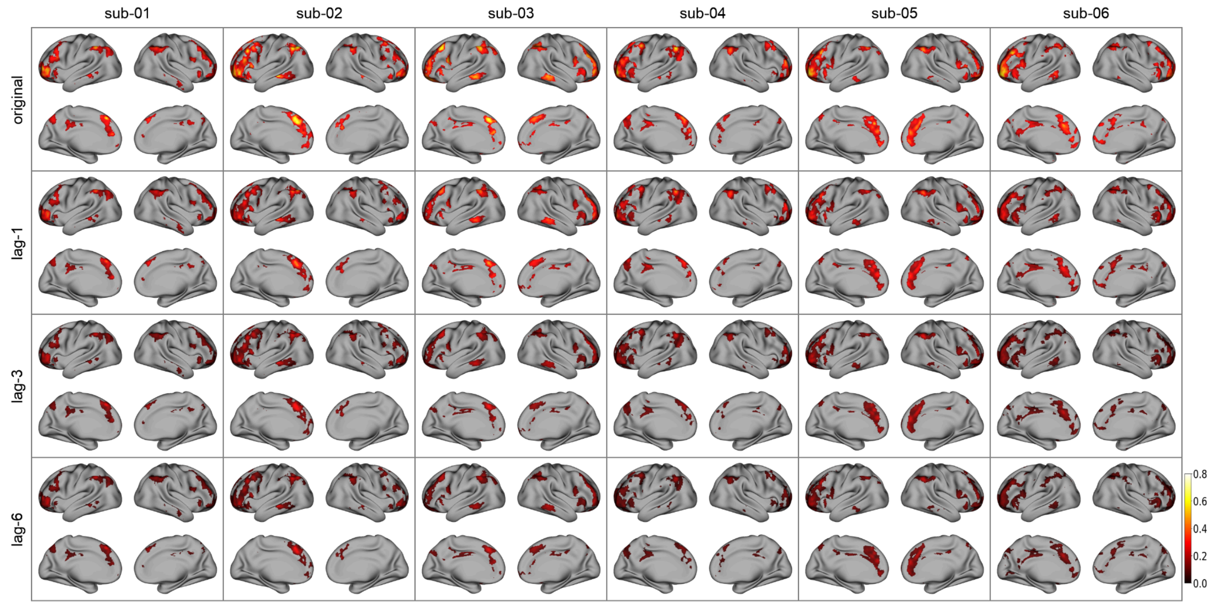

Fig II.5: Seed-based connectivity maps with a seed in the fronto-parietal network (MNI coordinates -40, 50, 7).

III: Data scaling of linear models

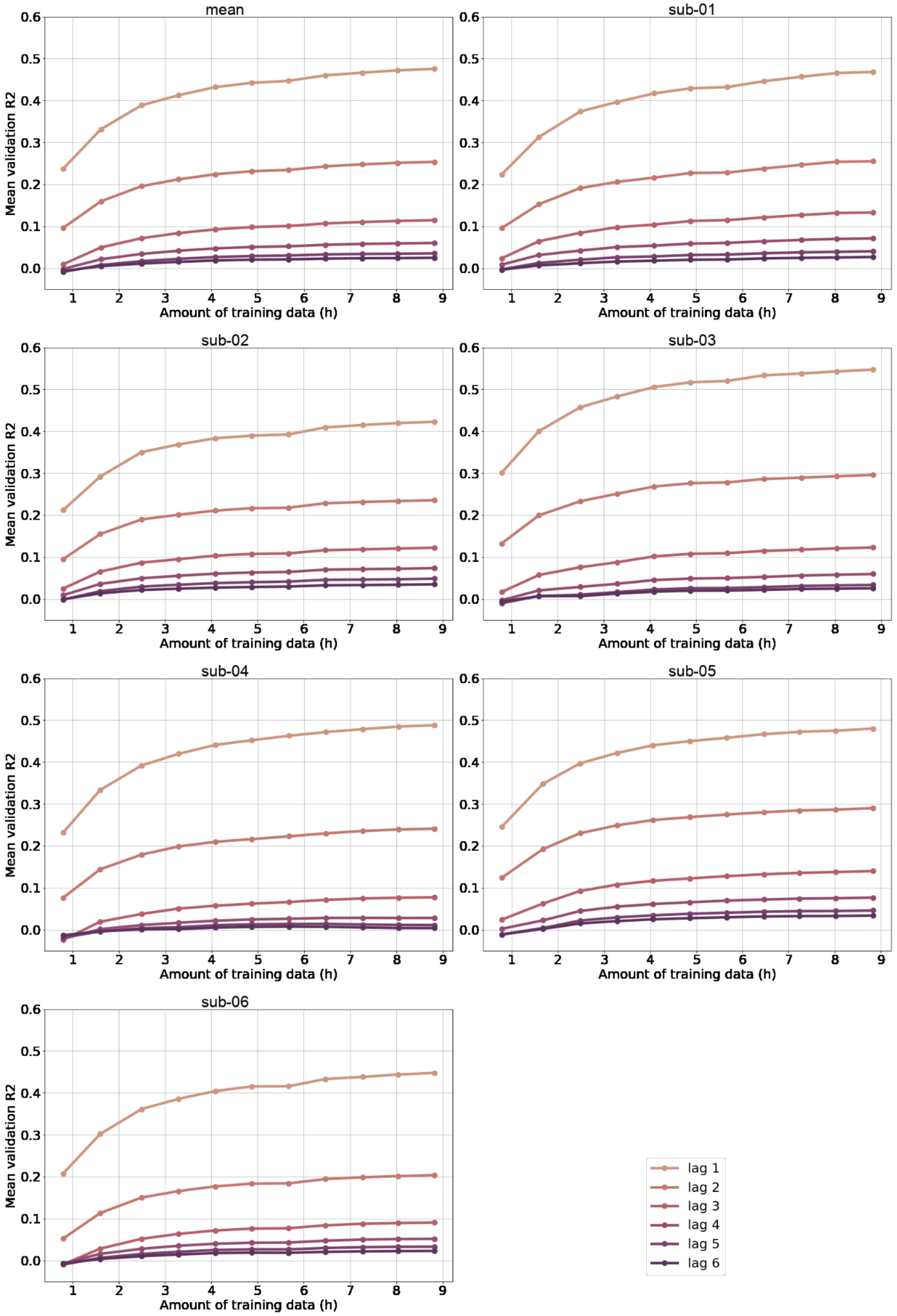

Fig III.1: Effect of training data amount on validation R^2 of the linear multivariate models.

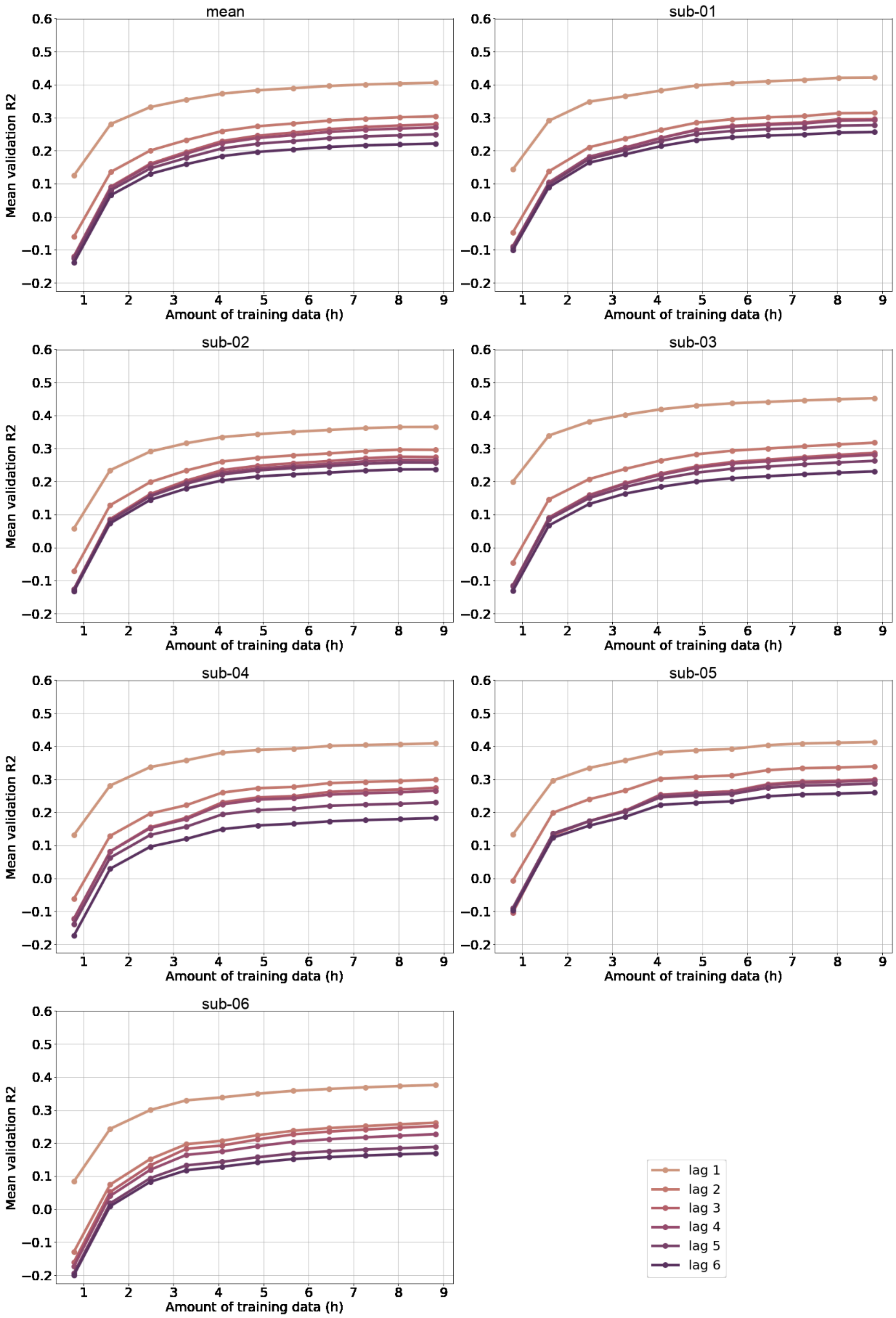

Fig III.2: Effect of training data amount on validation R² of the linear univariate models.

IV: Dataset versions

The specific commits of each of the datalad datasets used in this study are the following:
- friends (friends.fmriprep): commit e7378cd30e7389013c07bcab0b30416ff92e8910
- hcptrt (hcptrt.fmriprep): commit 4f1a2b1945b54741270e1036380eaaf0f0fb77cc
- movie10 (movie10.fmriprep): commit 6ab056448ac51f01c5fafe11a027d230c94d9997

V: Hyper-parameters

For each of the models, here are the hyper-parameters used in the gridsearch. The lists denote the different values tried for a parameter, with the optimal parameters in bold.

- **Chebnet**:
  seq_length [16, 64, 128, **192**, 256]
  time_stride [1]
  FK ['8,6,8,6,8,6', '16,3,16,3,8,3,8,3', '64,3,32,3', '16,3,8,3', '32,3', **'8,3,8,3,8,3'**, '1,1']
  M ['16,8,1', **'1'**, '8,1']
  FC_type ['nonshared_uni']
  edge_index_thres [0.9]
  dropout [0]
  bn_momentum [0.1]
  use_bn [True]
  nb_epochs [0, 12, 25, 50, 75, **100**]
  batch_size [100]
  lr [0.01]
  lr_patience [4]
  lr_thres [0.001]
  weight_decay [0]

- **LRuni**:
  seq_length [4, 16, 32, 64, 128, 192, **256**]
  time_stride [1]
  F ['1']
  dropout [0.1]
  bn_momentum [0.1]
  use_bn [True]
  nb_epochs [0, 12, 25, 50, 75, **100**, 120]
  batch_size [**100**, 500]
  lr [0.0001, **0.01**]
  lr_patience [4]
  lr_thres [0.001]
  weight_decay [0]

- **MLPuni**:
  seq_length [4, 16, 32, 64, **128**, 192, 256]
  time_stride [1]

F ['32,16,1', **'8,1'**, '8,8,1', '8,8,8,1']
dropout [0.1]
bn_momentum [0.1]
use_bn [True]
nb_epochs [0, 12, 25, 50, 75, **100**, 120]
batch_size [**100**, 500]
lr [0.0001, **0.01**]
lr_patience [4]
lr_thres [0.001]
weight_decay [0]

- **LRmulti**:
  seq_length [1, 2, **3**, 4, 8, 16]
  time_stride [1]
  F ['1']
  dropout [0.1]
  bn_momentum [0.1]
  use_bn [False, **True**]
  nb_epochs [0, 12, 25, 50, 75, 100, **120**]
  batch_size [800]
  lr [0.0001]
  lr_patience [4]
  lr_thres [0.001]
  weight_decay [0]

- **MLPmulti**:
  seq_length [1, 2, **3**, 4, 8, 16]
  time_stride [1]
  F [**'4,1'**, '8,1', '8,4,1', '8,8,4,1’]
  dropout [0.1]
  bn_momentum [0.1]
  use_bn [False, True]
  nb_epochs [0, 12, **25**, 50, 75, 100, 120]
  batch_size [800]
  lr [0.0001]
  lr_patience [4]
  lr_thres [0.001]
  weight_decay [0]

- **RNN**:
  horizon [6]
  input_chunk_length [8, 16, 32, **64**]
  hidden_dim [8, 16, **32**, 64]
  n_rnn_layers [1, 2, **4**]
  training_length [100]
  dropout [0.1]
  n_epochs [20, **60**, 100]
  batch_size [**100**, 800]

- **NBEATS**:
  input_chunk_length [**2**, 4, 8, 16, 32, 64]
  output_chunk_length [6]
  generic_architecture [True]
  num_stacks [30]
  num_blocks [**1**, 2]
  num_layers [**2**, 4]
  layer_widths [**16**, 64]
  expansion_coefficient_dim [2, **5**]
  n_epochs [**20**, 60, 100]
  batch_size [100, **800**]
  optimizer_kwargs ["{'lr': 0.01, 'weight_decay': 0}"]
  lr_scheduler_cls ['ReduceLROnPlateau']
  lr_scheduler_kwargs ["{'factor': 0.1, 'patience': 4, 'threshold': 0.001}"]

- **GRU**:
  horizon [6]
  input_chunk_length [8, 16, 32, **64**]
  hidden_dim [8, **16**, 32, 64]
  n_rnn_layers [1, **2**, 4]
  training_length [100]
  dropout [0.1]
  n_epochs [**20**, 60, 100]
  batch_size [**100**, 800]

- **LSTM**:
  horizon [6]
  input_chunk_length [8, 16, **32**, 64]
  hidden_dim [8, **16**, 32, 64]
  n_rnn_layers [1, **2**, 4]
  training_length [100]
  dropout [0.1]
  n_epochs [**20**, 60, 100]
  batch_size [**100**, 800]

- **TFT**:
  input_chunk_length [8, 16, **32**]
  output_chunk_length [6]
  hidden_size [16, **64**]
  lstm_layers [1]
  num_attention_heads [4]
  full_attention [False]
  hidden_continuous_size [8]
  add_relative_index [True]
  dropout [0.1]
  n_epochs [**20**, 60]
  batch_size [600]

- **Transformer**:
  input_chunk_length [**1**, 4, 8, 16]
  output_chunk_length [6]
  d_model [**64**, 128]
  nhead [4]
  num_encoder_layers [3]
  num_decoder_layers [3]
  dim_feedforward [**64**, 128]
  dropout [0.1]
  n_epochs [**20**, 60, 100]
  batch_size [**100**, 800]

VI Comparison between iterated single-step and multi step prediction scores for the Chebnets

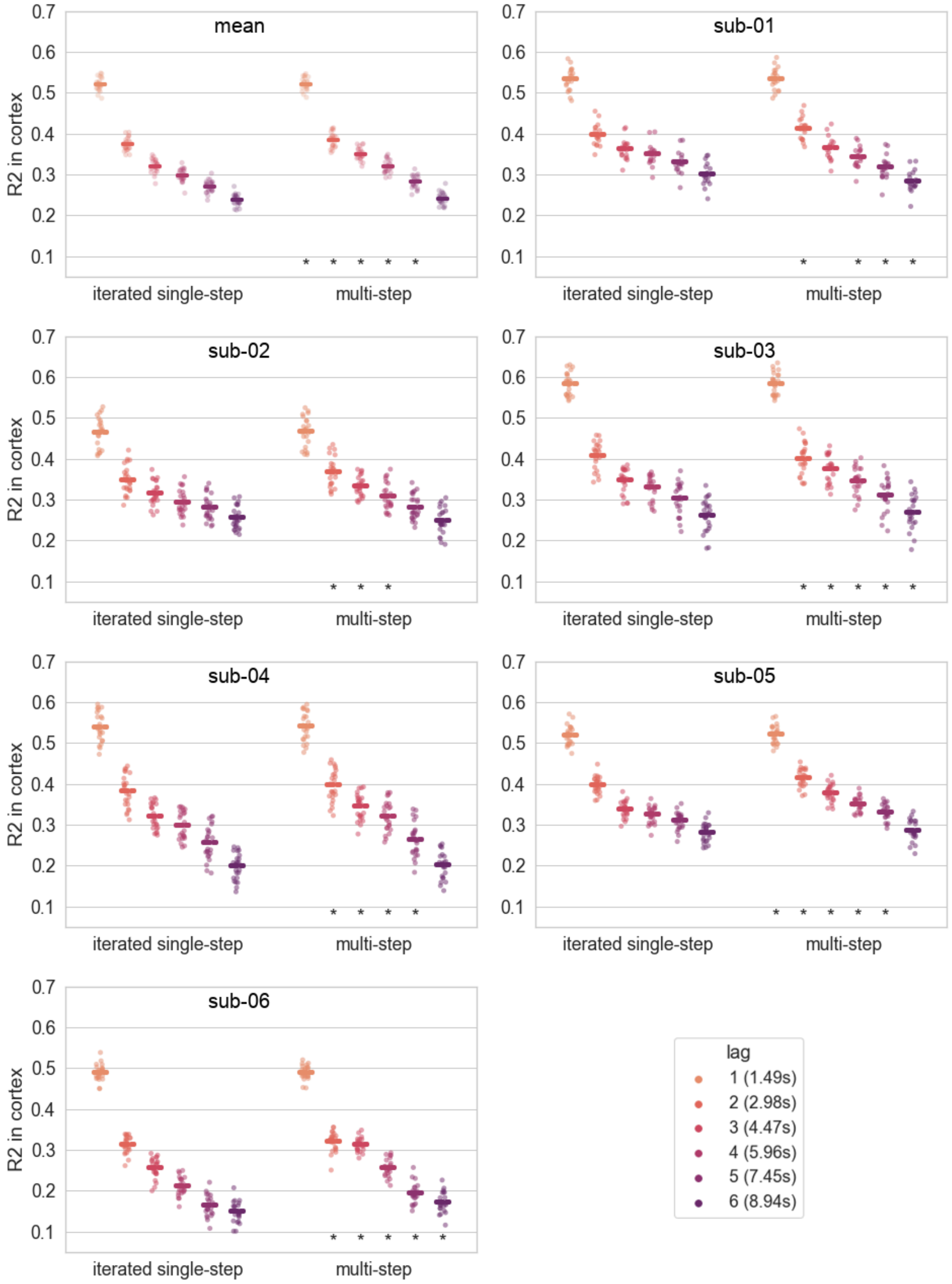

Figure VI.1 Comparison between the iterated single-step and multistep prediction methods for the Chebnets. The multi-step prediction shows overall significantly better performance, but with an improvement mostly negligible (~0.01) compared to the differences observed when varying the models or subjects (~0.1). The asterisks denote a significant difference between the scores of the multi-step and iterated single-step prediction methods for a given subject and predicted lag. The significance corresponds to a p-value < 0.05 for a two-sided Wilcoxon test.
